# Near-infrared light reduces glia activation and modulates neuroinflammation in the brains of diet-induced obese mice

**DOI:** 10.1038/s41598-022-14812-8

**Published:** 2022-06-27

**Authors:** Salvatore Saieva, Giulio Taglialatela

**Affiliations:** 1 Department of Neurology, Mitchell Center for Neurodegenerative Diseases, University of Texas Medical Branch, Galveston, TX USA; 2 Department of Neuroscience, Cell Biology and Anatomy, University of Texas Medical Branch, Galveston, TX USA

**Keywords:** Neuroimmunology, Neurotrophic factors, Neuroscience, Cellular neuroscience, Chronic inflammation, Cytokines, Inflammation, Neuroimmunology, Immunochemistry, RNA, Immunohistochemistry, Reverse transcription polymerase chain reaction, Fluorescence imaging, Immunohistochemistry, Imaging, Immunological techniques

## Abstract

Neuroinflammation is a key event in neurodegenerative conditions such as Alzheimer’s disease (AD) and characterizes metabolic pathologies like obesity and type 2 diabetes (T2D). Growing evidence in humans shows that obesity increases the risk of developing AD by threefold. Hippocampal neuroinflammation in rodents correlates with poor memory performance, suggesting that it contributes to cognitive decline. Here we propose that reducing obesity/T2D-driven neuroinflammation may prevent the progression of cognitive decline associated with AD-like neurodegenerative states. Near-infrared light (NIR) has attracted increasing attention as it was shown to improve learning and memory in both humans and animal models. We previously reported that transcranial NIR delivery reduced amyloid beta and Tau pathology and improved memory function in mouse models of AD. Here, we report the effects of NIR in preventing obesity-induced neuroinflammation in a diet-induced obese mouse model. Five-week-old wild-type mice were fed a high-fat diet (HFD) for 13 weeks to induce obesity prior to transcranial delivery of NIR for 4 weeks during 90-s sessions given 5 days a week. After sacrifice, brain slices were subjected to free-floating immunofluorescence for microglia and astrocyte markers to evaluate glial activation and quantitative real-time polymerase chain reaction (PCR) to evaluate expression levels of inflammatory cytokines and brain-derived neurotrophic factor (BDNF). The hippocampal and cortical regions of the HFD group had increased expression of the activated microglial marker CD68 and the astrocytic marker glial fibrillary acidic protein. NIR-treated HFD groups showed decreased levels of these markers. PCR revealed that hippocampal tissue from the HFD group had increased levels of pro-inflammatory interleukin (IL)-1β and tumor necrosis factor-α. Interestingly, the same samples showed increased levels of the anti-inflammatory IL-10. All these changes were attenuated by NIR treatment. Lastly, hippocampal levels of the neurotrophic factor BDNF were increased in NIR-treated HFD mice, compared to untreated HFD mice. The marked reductions in glial activation and pro-inflammatory cytokines along with elevated BDNF provide insights into how NIR could reduce neuroinflammation. These results support the use of NIR as a potential non-invasive and preventive therapeutic approach against chronic obesity-induced deficits that are known to occur with AD neuropathology.

## Introduction

Obesity and dementia are serious health conditions with growing incidence rates in the global population^1,2^. The last two decades have seen the publication of several studies suggesting that obesity is a significant risk factor in promoting progressive cognitive decline leading to dementia, including Alzheimer’s disease (AD)^3–5^. The most common form of dementia, AD is characterized by progressive loss of memory and cognitive decline^6,7^. From a neuropathologic point of view, AD is characterized by two major hallmarks: (1) extracellular deposition of amyloid-β (Aβ) with subsequent neuritic plaque formation and (2) aggregation of intraneuronal neurofibrillary tangles composed of hyperphosphorylated Tau^2,8^. Nonetheless, aggregate deposition occurs throughout several years before the overt appearance of clinical symptoms^9^. In fact, plaque and tangle formation is preceded by the formation of soluble oligomers of both Aβ and Tau, and these are considered the most toxic species driving the preclinical phase of AD^10–12^. Several risk factors (e.g., genetic mutations or co-morbidities) may accelerate AD onset and exacerbate disease progression^2,13,14^, indicating the involvement of multiple pathological mechanisms^15^. These include early events such as synapse loss, oxidative stress, and neuroinflammation^16,17^.

Sadly, no disease-modifying treatments are available for AD, and the few approved drugs provide only limited symptomatic relief^18^; in addition, pharmacological drugs targeting either Aβ or Tau have thus far proven ineffective in halting or reversing cognitive decline^19,20^. In the last decade, alternative approaches targeting AD risk factors have been proposed, such as physical exercise, antioxidant supplements, healthy diets, and nutraceutical use^21,22^, all aimed at reducing the incidence of AD risk factors.

Obesity is one of the 12 most prominent risk factors for AD that are considered modifiable^23,24^. Thus, preventing obesity-associated pathological states could slow or stop progression to dementia and therefore delay or prevent AD^24–26^. Obesity is a medical condition defined as excessive accumulation of adipose tissue that may pose serious threats to life due to an imbalance between energy intake and energy expenditure^1^. It has a multifactorial pathogenesis, due to genetic, lifestyle, environmental and familiar factors and is strongly associated with other co-morbidities such as type 2 diabetes mellitus (T2DM), cardiovascular diseases, and neurodegenerative disorders^27,28^. In this study, we focused on a specific type of obesity induced by a high-fat diet (HFD).

Independent of other comorbidities, several studies have established an association between obesity and dementia. Compelling evidence has established that mid-life obesity correlates with lower cognitive performance and increased AD risk^29,30^. Researchers have proposed several pathological mechanisms underlying this association^31–36^; among them, neuroinflammation has attracted increasing interest in recent years.

Neuroinflammation is a defense mechanism that protects the brain from infection or injury via activation of tissue repair and debris removal mechanisms^37^. However, prolonged neuroinflammation can damage the central nervous system (CNS) leading to neurodegeneration^38^ and is also present in AD, especially in the early stages^37^. Evidence suggests that Aβ aggregates trigger an immune response in microglia and astrocytes^39^ characterized by morphological changes and increased production and release of pro-inflammatory molecules. Once activated, microglia and astrocytes can phagocytize Aβ aggregates to remove them from the CNS^40^. However, the phagocytic process is rendered ineffective in AD, thus favoring Aβ^41^ spread and aggregation and exacerbating excessive production and release of pro-inflammatory factors^42^. The downstream effects of these events include increased blood–brain barrier (BBB) permeabilization, higher leukocyte infiltration, and activation of pro-inflammatory pathways that perpetuate aggregation and neurodegeneration^38,43^.

Obesity causes a systemic low-grade inflammatory state characterized by excessive circulation of free fatty acids and release of inflammatory cytokines secreted by peripheral organs^44,45^. HFD-induced obesity also leads to neuroinflammation^46^; the massive concentration of free fatty acids (which are able to cross the BBB^47^) enables their binding to specific microglia receptors (e.g., Toll-like receptor-4 [TLR4]), which activates microglia and increases the release of pro-inflammatory cytokines^29^. Chronic HFD consumption leads to neuroinflammation and likely to neurodegeneration^25,46,48^. Notably, short-term HFD in rodent models of obesity show hypothalamic dysfunction and neuroinflammation just in 3 days^49^, while longer HFD regimens (> 2 weeks) show neuroinflammation in other brain regions such as the hippocampus, amygdala, and frontal cortex (FCTX)^50,51^. The effects of HFD-induced neuroinflammation in the hippocampus involve impaired synaptic plasticity that precedes cognitive deficits^26^. HFD-induced neuroinflammation in the hippocampus of diet-induced obese (DIO) wild-type mice triggers Aβ and Tau deposition^52^ and drives behavioral deficits and an AD-like phenotype^33,34^. Another interesting aspect is the effect of an HFD on brain-derived neurotrophic factor (BDNF). Rodent models of obesity exhibit a downregulation of BDNF with consequent impairment of cognitive function, especially in the hippocampus where BDNF’s contribution to synaptic plasticity and memory processes is prominent^53–55^. BDNF also seems to have an important role in energy metabolism by reducing food intake, therefore HFD-induced BDNF reduction leads to dysregulated food intake and ultimately to weight gain and possibly obesity^56,57^.

In the present study, we propose a novel non-invasive strategy that was recently developed and proposed as potential treatment for several CNS pathologies: near-infrared light (NIR), also referred to as photobiomodulation (PBM) or low-level laser therapy^58,59^. PBM use has been published several times in the last 30 years; it comprises the electromagnetic radiations that fall in the range between 600 and 1100 nm, with power density below 500 W/cm^2^ and energy between 1 and 20 J/cm^2^^60,61^. NIR lasers have good penetration through skin and soft/hard tissue without heating (hence cold lasers), making them suitable for pain relief, wound healing, tissue regeneration, and as anti-inflammatory treatments^60^.

This strategy has shown beneficial effects in several rodent models of neurodegenerative disease such as stroke^62^, sleep deprivation^63^, major depressive disorder^64^, traumatic brain injury (TBI)^65^, multiple sclerosis^66^, spinal cord injury (SCI)^60^ and Parkinson’s disease (PD)^67,68^. Our group and others have demonstrated that NIR protects synapses from binding toxic Aβ oligomers and improves cognitive function in different AD-like mouse models^69–72^. In the present study, we investigated the effects of NIR on HFD-induced changes in the hippocampus and cortex: neuroinflammation, increased glia activation and pro-inflammatory cytokine expression, and downregulation of the neurotrophic factor BDNF. We demonstrate that NIR reduces the impact of obesity on brain function, particularly with regard to neuroinflammation, one of the key events underlying dementia. Combining our previous results in AD-like mouse models^69,70^ with these results suggests that this approach has potential to be developed as a novel, preventative, and non-invasive treatment for people with an obesity-related risk of developing dementia.

## Methods

### Animals and diet

The experimental design is summarized in Supplementary Fig. S1. Male C57Bl/6 J mice were utilized in this study. We employed male mice in this study, on the basis that previous literature (reviewed in^73^) has unequivocally reported that female mice often fail to show alterations of glucose tolerance and neuroinflammation in response to HFD^74,75^. The mice were purchased from Jackson Laboratory (cat #000664, Bar Harbor, ME, USA) at 4 weeks of age; after 1 week of acclimation, the mice were randomly divided in two groups (n = 10/group), and fed ad libitum with either regular chow (RC, cat #7012, Teklad, Madison, WI, USA, see Table 1 for diet composition), or an HFD (cat. #D12492, Research Diet, New Brunswick, NJ, USA, see Table 1 for composition) for 17 consecutive weeks. The mice were weighed once a week. At week 13 after the beginning of the diet, the mice were further subdivided in four experimental groups (n = 5/each): RC-fed not treated with light (RC SHAM), RC-fed treated with NIR (RC NIR), HFD-fed not treated with light (HFD SHAM), and HFD-fed treated with NIR (HFD NIR). After the end of NIR treatments described below, all the mice were euthanized by exposure to isoflurane, perfused with 1X phosphate-buffer saline (PBS) and decapitated. The brains were quickly removed, and the two hemispheres were separated: one was further dissected into major regions (FCTX, hippocampus, parietal/occipital cortex [POCTX], midbrain, cerebellum) and stored at − 80 °C until further analysis; the other was post-fixed with formalin (cat #245-684, Thermo Fisher Scientific, Waltham, MA, USA) for 48 h at 4 °C, after which the brain were transferred in 1X PBS + 0.01% sodium azide at 4 °C and shipped to Neuroscience Associates (Knoxville, TN, USA) for tissue processing. All experimental protocols were approved by the Institutional Animal Care and Use Committee of the University of Texas Medical Branch (UTMB). All methods were performed in accordance with the guidelines and regulations of the committee. Animals were housed under USDA standards (12:12-h light:dark cycle, food and water ad libitum) at the UTMB vivarium.

**Table 1:** Diet composition.

Nutrient composition	Regular chow (Teklad, 7012)	High-fat diet (Research Diet, D12492)
Protein	19.1% kcal	20% kcal
Fat	5.8% kcal	60% kcal
Carbohydrate	44.3% kcal	20% kcal
Energy density	3.1 kcal/g	5.21 kcal/g

Fat composition	Regular chow (%)	High-fat diet (%)
C16:0 palmitic	0.6	49.9
C18:0 stearic	0.2	26.9
C18:1ω9 oleic	1.3	86.3
C18:2ω6 linoleic	2.6	72.7
C18:3ω3 linolenic	0.3	5.1
Total saturated	0.8	32.2
Total monounsaturated	1.3	35.9
Total polyunsaturated	2.9	31.9

The table shows the differences in nutrient composition between the regular diet and the HFD, with a focus on the fat composition for each diet.

### Intraperitoneal glucose tolerance test (IPGTT)

IPGTT was performed 12 weeks after HFD initiation as previously described^76^. Briefly, glucose (cat #G8644, Sigma-Aldrich, St. Louis, MO, USA) was administered by intraperitoneal injection at a dose of 1 g/kg body weight following a 5-h fast. Blood samples were obtained via the tail vein at baseline (0 min) and 15, 30, 60, 90, and 120 min after injection. Blood glucose levels were measured using a Contour next glucometer (Ascensia Diabetes Care, Parsippany, NJ, USA).

### NIR treatment

Thirteen weeks following the start of HFD feeding, we started NIR treatment as we previously described^69,70^. The treatment groups (RC NIR and HFD NIR) received 1 dose per day, 5 days a week, for 4 consecutive weeks. One dose consisted of a 90-s treatment from a light-emitting diode device (WARP 10; Quantum Devices, Barneveld, WI, USA) with a wavelength of 670 nm and energy equal to 4 J/cm^2^ per treatment. Each mouse was hand restrained, and the head was placed approximately 1 cm from the light source. The body of each mouse was covered with aluminum foil to prevent light exposure to the periphery. The control groups (RC SHAM and HFD SHAM) were restrained in the same way and for the same amount of time, but the device was kept off.

### Free-floating immunofluorescence (FrFl-IF)

The brain samples were processed by the company Neuroscience Associates using patented MultiBrain® technology. Briefly, the 20 mouse brain hemispheres were embedded together in a gelatinous block and freeze-sectioned at 35-µm in the coronal plane through the cerebrum. All sections were collected into a series of 24 cups containing antigen preserve solution. After sectioning, the cups containing the sections were shipped back to our lab for immunostaining. FrFl-IF was performed as previously described^77^ with minor modifications. Briefly, non-specific binding sites were blocked with 5% bovine serum albumin (BSA, cat #A4503-100G, Sigma-Aldrich)/10% normal goat serum (NGS, cat #S-1000, Vector Laboratories, Burlingame, CA, USA) in 1X Tris-buffered saline (TBS), and the sections were permeabilized with 0.5% Triton X-100 and 0.05% Tween-20 for 1 h at room temperature. The slices were then incubated with the following primary antibodies containing 1.5% NGS in 1X TBS, overnight at 4 °C: rabbit anti-ionized calcium-binding adapter-1 (Iba-1, dilution 1:200, cat #019-19,741, Wako, Fujifilm Wako Pure Chemicals Corporation, Osaka, Japan), chicken anti-glial fibrillary acidic protein (GFAP, dilution 1:500, cat #GFAP, Aves Labs, Davis, CA, USA), rat anti-cluster of differentiation 68 (CD68, dilution 1:500, cat #ab53444, Abcam, Cambridge, UK), NLR [NOD (Nucleotide-binding Oligomerization Domain-like) Like Receptor] family Pyrin domain containing 3 (NLRP3, dilution 1:100, cat #NBP2-12,446, Novus Biologicals, Centennial, CO, USA). After washing with 1X TBS, the slices were incubated with the appropriate Alexa-conjugated secondary antibodies (goat anti-rabbit Alexa Fluor 488, 1:400, cat #A-11008; goat anti-chicken Alexa Fluor 594, 1:400, cat #A-11042; goat anti-rat Alexa Fluor 594, 1:400, cat #A-11007, Invitrogen, Carlsbad, CA, USA) in 1X TBS containing 1.5% NGS. After washing with 1X TBS, the slices were adhered onto glass slides (50 × 76 × 1.2 mm) and air-dried. Finally, the slices were treated with 0.3% Sudan Black in 70% ethanol for 10 min to remove autofluorescence, briefly washed in deionized H_2_O, coverslipped using Fluoromount-G containing 4′,6-diamidino-2-phenylindole (DAPI, cat #0100-20, SouthernBiotech, Birmingham, AL, USA), and sealed.

### Microscopy and image analyses

All images were acquired with a Keyence BZ-X800 (Keyence Corporation, Osaka, Japan) microscope using 40X and immersion oil 60X objectives. From each of the two analyzed sections, we analyzed three images from each hippocampal subregion (CA3, CA1, and DG); 4 images from the POCTX; and five images from the FCTX. Quantitative analysis was performed using ImageJ software (https://imagej.nih.gov/ij, National Institutes of Health, Bethesda, MD, USA) by analyzing the fluorescence intensity of each marker (Integrated Density, IntDen). The expression of the chosen markers was quantified by measuring the average fluorescence intensity—and for GFAP staining, by measuring also the percentage of stained area—of each analyzed area. A threshold for positive staining was determined for each image that included all cell bodies and processes but excluded background staining. Moreover, a manual count for microglial cells both CD68-positive and Iba-1-positive (CD68^+^/Iba-1^+^) was performed. Representative images were composed in CC2021 format in Adobe Photoshop (San Jose, CA, USA).

### Morphologic analyses of microglia

To perform morphological analyses of microglia, we used the software Imaris 9.7.2 (Oxford Instruments, Concord, MA, USA). The TIFF images obtained by Keyence were opportunely converted in IMS format to be used in Imaris environment. To measure the area covered by the microglia cell body we used the feature “Surface”; for all the images analyzed, we set the parameter Surfaces Detail at 0.250 μm (smooth), while thresholding background subtraction (local contrast). Moreover, we used the filter function to remove unspecific background signals, although we manually removed those microglia with incomplete cell bodies. In addition, we applied the “filter/area function” to remove small microglial segments. After deletion of all background signals, the “mask all” function was used to create the final surface reconstruction.

For the measurements of filament area, filament length, number of branch points and number of segments, we used the tool “Filament Tracer” by analyzing 5 microglial cells per each brain area we analyzed, for a total of 25 cells per animal. First, we drew a region of interest, on which we applied the “Surface” tool as previously described, followed by the Filament Tracer set on the algorithm “Autopath (no loops)”. For all the microglia we set seed points in the range 0.185-0-195 µm. while the largest diameter was depending on the size of the cell (specifically, we measured the diameter of each “microglia sphere” with the function “Slice”). The software was instructed to detect starting points and the seed points were manually corrected to either include portions of microglia filament not initially detected or to remove filaments that were wrongly identified. Also, we made sure that the seed points around the starting point were removed. The final algorithm was set on the shortest distance from distance map.

All surface and filament parameters were exported into separate Excel files and used for data analysis.

### Quantitative real-time polymerase chain reaction (q-RT-PCR)

Total RNA from the hippocampus and FCTX was isolated using RNeasy® Mini kits (cat #74,104, Qiagen, Hilden, Germany). Briefly, approximately 50 mg of tissue were homogenized and processed following the manufacturer’s protocol, and the samples were resuspended in 50 µl nuclease-free water. The RNA concentration was measured using NanoDrop 2000c (Thermo Fisher Scientific). q-RT-PCR was performed to quantify mRNA in the RC SHAM, RC NIR, HFD SHAM and HFD NIR mice. RNA samples (30 ng) were mixed with QuantiFast® SYBR® Green RT-PCR Kit (cat #204,154, Qiagen) and QuantiTect® Primer Assay (product #249900, Qiagen-tumor necrosis factor [TNF]-α, cat #QT00104006-interleukin [IL]-1β, cat #QT01048355-IL-10, cat #QT00106169-BDNF cat #QT00097118-β-actin, cat #00095242) following the manufacturer’s protocol and their suggested cycler conditions. The reaction was performed in Mastercycler epgradient S (Eppendorf, Hamburg, Germany). TNF-α, IL-1β, IL-10, and BDNF mRNA levels were normalized to those of β-actin. The relative fold changes in target miRNA expression were determined using the comparative cycle threshold method (2^−ΔΔCt^).

### Statistical analyses

Statistical analyses were performed using GraphPad Prism 8.4.3 and 9.3.1 software (GraphPad Inc., San Diego, CA, USA). Significant differences between groups were determined with Student’s t-tests with Mann–Whitney post-hoc tests, one-way analyses of variance (ANOVAs) with Tukey’s post hoc tests, or two-way ANOVAs with Sidak’s multiple comparison test. Data are expressed as mean ± SD, and *p* < 0.05 was considered statistically significant for all analyses.

### Ethics approval and consent to participate

This study was carried out in accordance with the recommendations in the Guide for the Care and Use of Laboratory Animals of the National Institutes of Health. All animal procedures were performed in compliance with the Institutional Animal Care and Use Committee at the University of Texas Medical Branch (UTMB) in Galveston, TX. The study was conducted in accordance with ARRIVE guidelines.

## Results

### Effect of HFD on body weight and glucose tolerance

We fed 10 mice with a 60% kcal HFD and 10 with an RC (control) ad libitum for 13 weeks before starting NIR treatments, and for additional 4 weeks during the time of the NIR treatment (see Supplementary Fig. S1 for experimental design). To establish whether the HFD altered metabolism, we measured changes in body weight (Supplementary Fig. S2A) and blood glucose levels (Supplementary Fig. S2B and S2C). A progressive difference in body weight was observed from week 1. The mice were weighed once a week; there was no significant change in body weight between the groups at baseline. After week 1, HFD-fed mice had higher body weight compared to RC-fed mice (Supplementary Fig. S2A). The difference in body weight became more evident over time and plateaued by week 11. Twelve weeks after HFD initiation, IPGTTs were performed to assess the effect of the diet on glucose utilization^78^. The test showed no difference in blood glucose levels between RC- and HFD-fed mice at minutes 0 and 15, but from 30 through 120 min, glucose levels were higher in the blood of HFD-fed mice (Supplementary Fig. S2B), compared to the RC group (confirmed by areas under the curve in Supplementary Fig. S2C).

### Effect of NIR on HFD-induced glia activation in the different brain areas of DIO mice

To evaluate whether obesity-induced glial activation is affected by NIR treatment, we analyzed the immunoreactive levels of Iba-1^79,80^ (microglia marker), CD68 (detects activated microglia^80^), and GFAP (astrocyte marker)^81^. We focused our attention on the hippocampus and cortex, two areas highly impacted by HFD/obesity-induced neuroinflammation in both human and rodents^26,51,82^. For the hippocampus, we separately analyzed the cornu ammonis 3 (CA3), cornu ammonis (CA1), and dentate gyrus (DG), while for the cortex we independently analyzed the FCTX and POCTX.

#### Hippocampus

**Figure 1:**
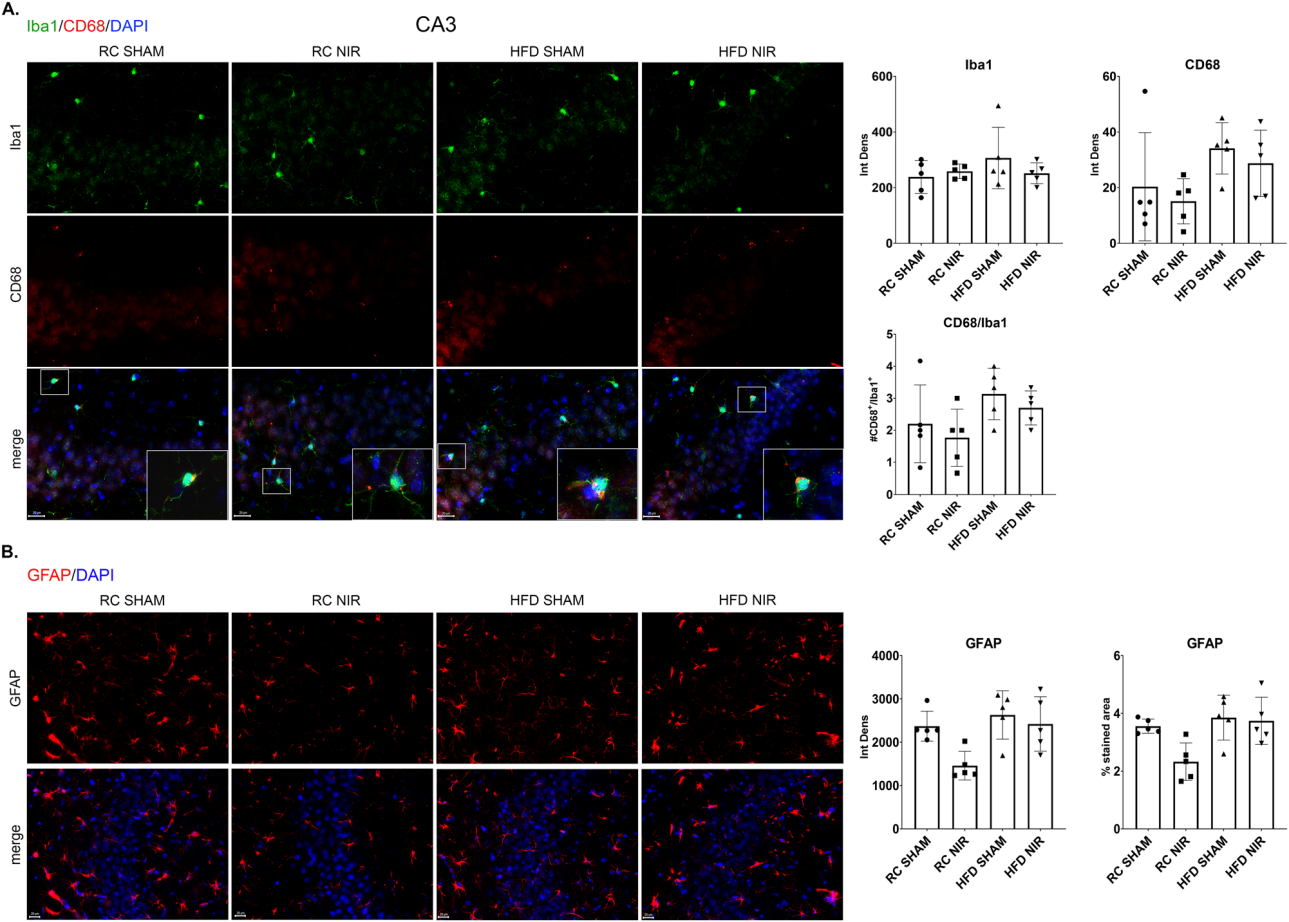
Glia marker expression in the CA3 of RC- and HFD-fed mice with or without NIR treatment. (**A**) Quantitative analyses of Iba-1 and CD68 showed no significant difference in microglia marker labeling in the hippocampal CA3. Magnification: 60X. (**B**) Quantitative analyses showed no change in GFAP labeling in the hippocampal CA3. Magnification: 40X. Image results of Z-stacks with pitch of 2.0 μm. n = 5 animals/group. Data are presented as mean ± SD. Each point is the average of 6 images/animal from 2 technical replicates. Statistical analyses: one-way ANOVA with Tukey’s post-hoc test. Single images were generated with Keyence BZ-X800 Analyzer (www.keyence.com). Graphs were created with GraphPad Prism 8.4.3 and 9.3.1 software (https://www.graphpad.com/). Images and graphs were composed in Adobe Photoshop 22.2.0 (https://www.adobe.com/products/photoshopfamily.html).

In the hippocampal CA3, quantification of the immunoreactive levels of Iba-1 (Fig. 1A), CD68 (Fig. 1A), and GFAP (Fig. 1B) did not show any significant differences among the four groups of mice with or without NIR treatment. Therefore, glia activation was neither induced by HFD nor modulated by NIR in the CA3.

**Figure 2:**
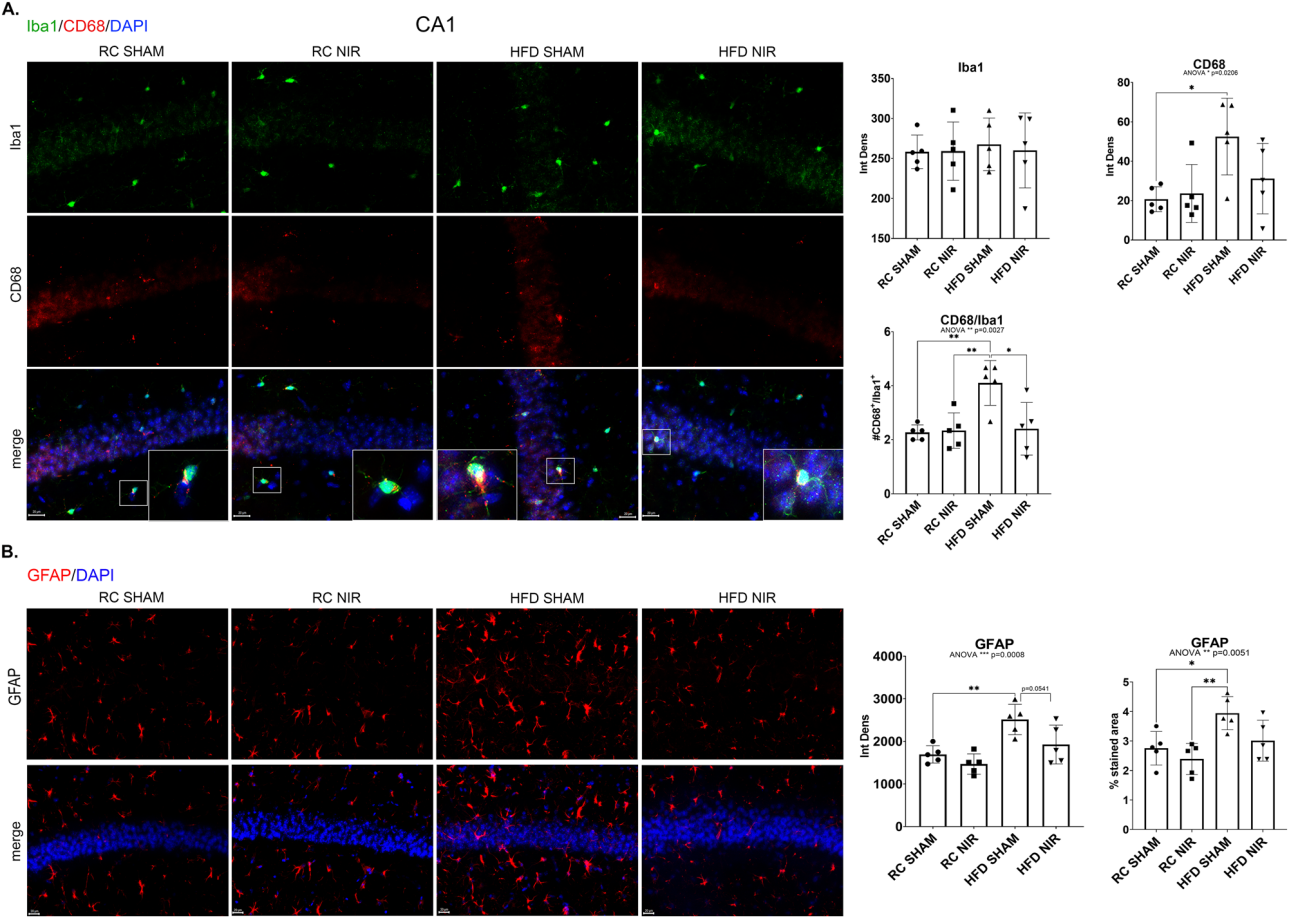
Glia marker expression in the CA1 of RC- and HFD-fed mice with or without NIR. (**A**) Iba-1 levels were similar across groups, while CD68 labeling showed increased microglia activation in the HFD SHAM versus control groups, and decreased levels after NIR treatment. Magnification: 60X. (**B**) GFAP immunoreactivity in the CA1 increased in the HFD regimen mice with respect to RC-fed mice, but this was normalized by NIR (*p* = 0.0541). Magnification: 40X. Image results of Z-stacks with pitch of 2.0 μm. n = 5 animals/group. Data are presented as mean ± SD. Each point is the average of 6 images/animal from 2 technical replicates. Statistical analyses: one-way ANOVA with Tukey’s post-hoc test. *p < 0.05, **p < 0.01. Single images were generated with Keyence BZ-X800 Analyzer (www.keyence.com). Graphs were created with GraphPad Prism 8.4.3 and 9.3.1 software (https://www.graphpad.com/). Images and graphs were composed in Adobe Photoshop 22.2.0 (https://www.adobe.com/products/photoshopfamily.html).

While Iba-1 levels were not different among the groups in the CA1 (Fig. 2A–B), CD68 levels significantly increased following HFD consumption compared to control animals (Fig. 2A). CD68 immunoreactivity was also reduced (*p* = 0.0541) in HFD mice treated with NIR treated compared with their untreated counterparts, suggesting that HFD-induced microglia activation in the CA1 is lowered by NIR. GFAP in the CA1 showed a similar trend (Fig. 2B), with an increase after HFD feeding compared to controls, that was reduced after NIR treatment. This evidence suggests that HFD-induced glia activation in the CA1 can be alleviated by NIR treatment.

**Figure 3:**
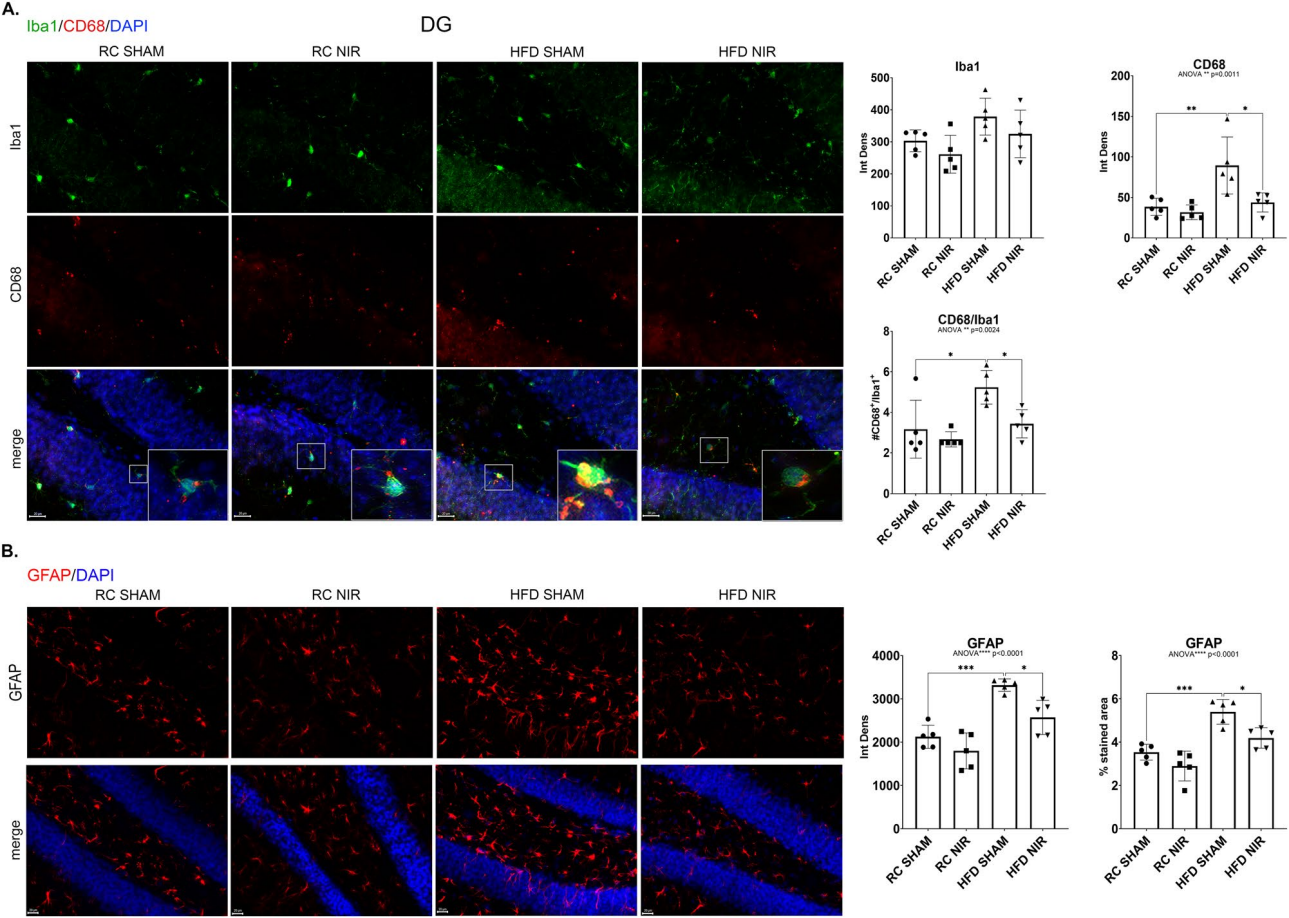
Glia marker expression in the DG of RC- and HFD-fed mice with or without NIR. (**A**) Iba-1 levels were not significantly different among the groups; CD68 was significantly elevated in the HFD group, but this was reversed by NIR. Magnification: 60X. (**B**) In the DG, NIR reduced HFD-induced astrocyte activation (GFAP immunoreactivity). Magnification: 40X. Image results of Z-stacks with pitch of 2.0 μm. n = 5 animals/group. Data are presented as mean ± SD. Each point is the average of 6 images/animal from 2 technical replicates. Statistical analyses: one-way ANOVA with Tukey’s post-hoc test. *p < 0.05, **p < 0.01, ***p < 0.001. Single images were generated with Keyence BZ-X800 Analyzer (www.keyence.com). Graphs were created with GraphPad Prism 8.4.3 and 9.3.1 software (https://www.graphpad.com/). Images and graphs were composed in Adobe Photoshop 22.2.0 (https://www.adobe.com/products/photoshopfamily.html).

We observed similar results in the DG (Fig. 3A–B). While Iba-1 did not show significant changes, CD68 did show a dramatic increase in HFD-fed mice compared to control mice and a return to normal after NIR treatment (Fig. 3A). Similarly, GFAP immunoreactivity was elevated in HFD mice compared to control mice and was lowered to normal levels following NIR compared to HFD SHAM (Fig. 3B). This demonstrates that NIR can also mitigate HFD-induced glia activation in the DG.

**Figure 4:**
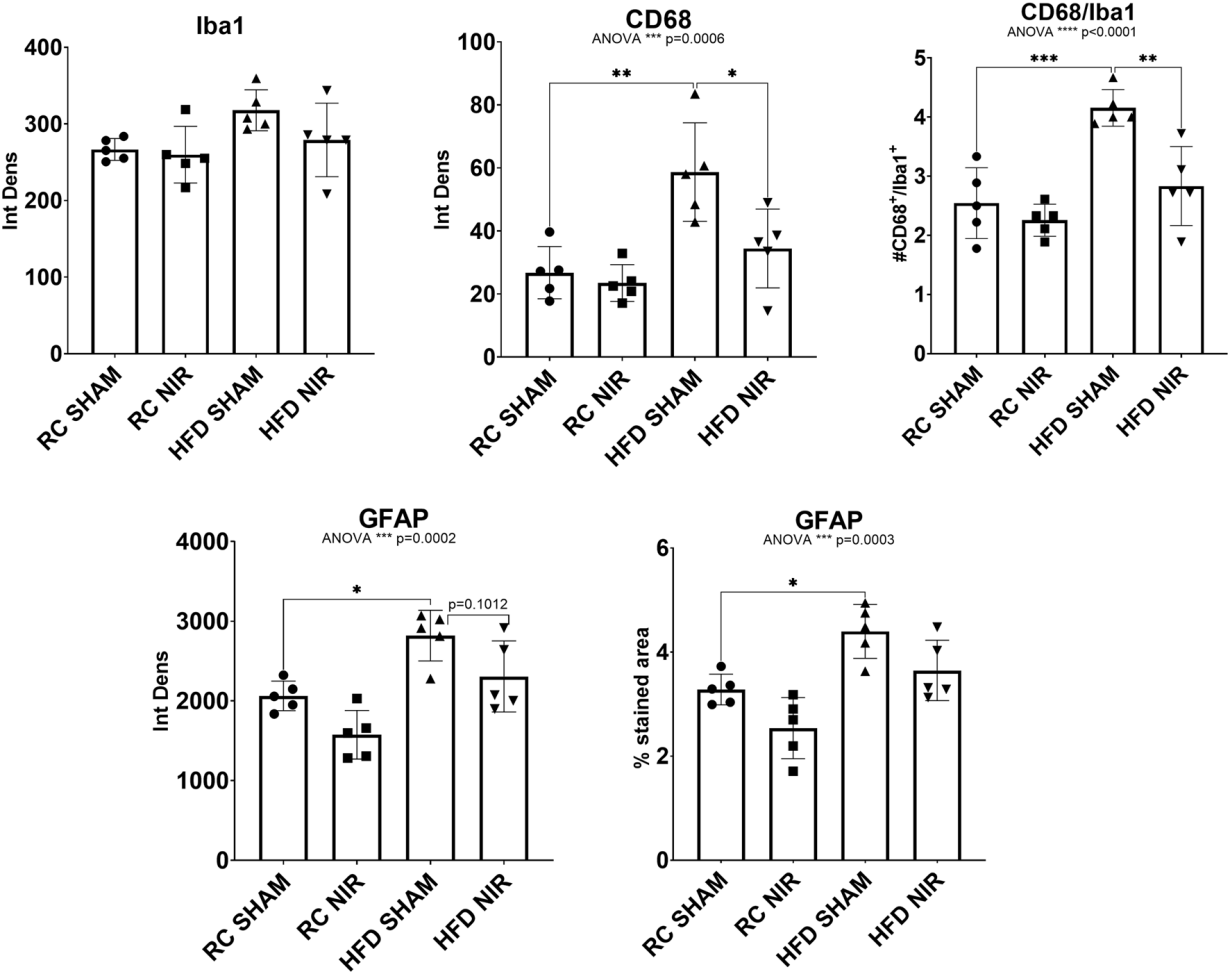
Glia marker expression in the entire hippocampi of RC- and HFD-fed mice with or without NIR. (**A**) The overall analyses of all hippocampal immunofluorescence images confirmed no significant differences in Iba-1 immunoreactivity among groups. (**B**) CD68 was increased in the HFD SHAM group, compared to control mice, but it decreased to levels close to RC-fed mice after NIR treatment. (**C**) GFAP was also significantly increased in the HFD SHAM group compared to control, but there is no significant difference between the HFD NIR and HFD SHAM groups. Data are presented as mean ± SD. Each point is the average of 18 images/animal from 2 technical replicates Statistical analyses: one-way ANOVA with Tukey’s post-hoc test. *p < 0.05, **p < 0.01, ***p<0.001. Graphs were created with GraphPad Prism 8.4.3 and 9.3.1 software (https://www.graphpad.com/). Images and graphs were composed in Adobe Photoshop 22.2.0 (https://www.adobe.com/products/photoshopfamily.html).

At the end of the analyses of each hippocampal area, we collected all the data from the CA3, CA1 and DG and performed a one-way ANOVA with Tukey’s post-hoc test (Fig. 4). Only CD68 showed significant reductions between the HFD SHAM and HFD NIR groups, while GFAP had a decreasing trending. Therefore, NIR mitigates hippocampal glia activation induced by an HFD.

The increased glia activation due to HFD feeding that took place in the DG and CA1 was reduced by NIR, while there were no changes in the CA3 induced by either HFD or NIR.

#### Cortex

**Figure 5:**
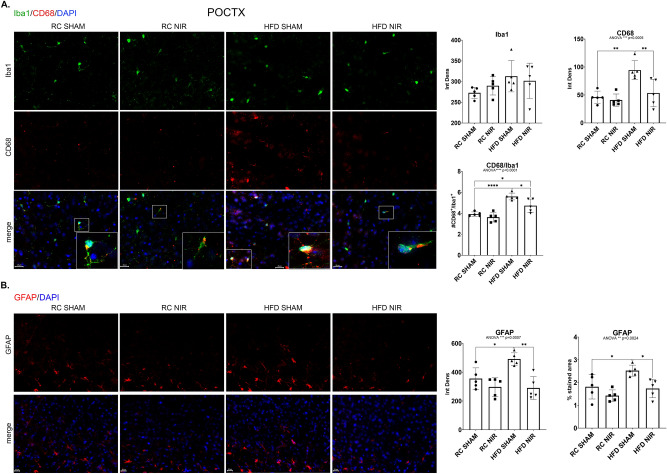
Glia marker expression in the POCTX of RC- and HFD-fed mice with or without NIR. (**A**) There were no significant differences in Iba-1 expression among the groups, but there was a dramatic increase of microglia activation (CD68) in HFD SHAM mice compared to control groups. NIR reduced CD68 in HF-fed mice. Magnification: 60X. (**B**) NIR reversed the HFD-induced increase in GFAP to levels close to control. Magnification: 40X. Image results of Z-stacks with pitch of 2.0 μm. n = 5 animals/group. Data are presented as mean ± SD. Each point is the average of 6 images/animal from 2 technical replicates. Statistical analyses: one-way ANOVA with Tukey’s post-hoc test. *p < 0.05, **p < 0.01, ****p<0.0001. Single images were generated with Keyence BZ-X800 Analyzer (www.keyence.com). Graphs were created with GraphPad Prism 8.4.3 and 9.3.1 software (https://www.graphpad.com/). Images and graphs were composed in Adobe Photoshop 22.2.0 (https://www.adobe.com/products/photoshopfamily.html).

**Figure 6:**
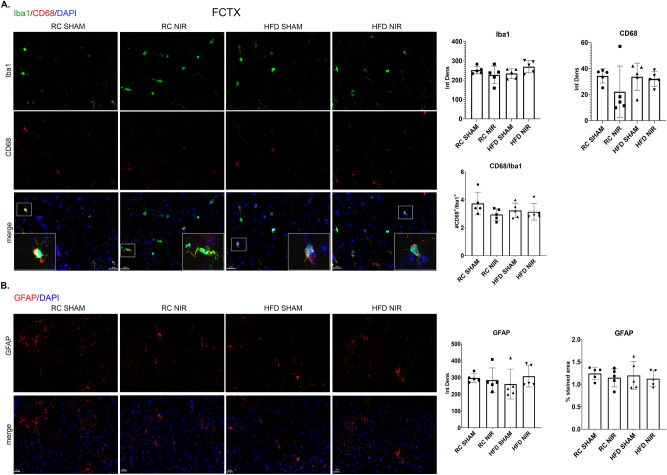
Glia marker expression in the FCTX of RC- and HFD-fed mice with or without NIR. (**A**) Quantitative analyses of microglia markers in the FCTX did not show any significant differences in Iba1 or CD68 among groups. Magnification: 60X. (**B**) GFAP immunoreactivity was similar in all groups. Magnification: 40X. Image results of Z-stacks with pitch of 2.0 of μm. n = 5 animals/group. Data are presented as mean ± SD. Each point is the average of 6 images/animal from 2 technical replicates. Statistical analyses: one-way ANOVA with Tukey’s post-hoc test. Single images were generated with Keyence BZ-X800 Analyzer (www.keyence.com). Graphs were created with GraphPad Prism 8.4.3 and 9.3.1 software (https://www.graphpad.com/). Images and graphs were composed in Adobe Photoshop 22.2.0 (https://www.adobe.com/products/photoshopfamily.html).

The POCTX showed trends very similar to the hippocampus (Fig. 5A–B). HFD induced more microglia (Fig. 5A) and astrocyte (Fig. 5B) activation in the HFD SHAM group that was attenuated in the HFD NIR group. The FCTX (Fig. 6A–B) did not show any changes caused by HFD or NIR, suggesting that this region reacted differently to the challenge from HFD.

### Morphologic analyses of microglia

An important feature of neuroinflammation is the morphological changes of microglia in response to stressors^39,83^. Accordingly, we investigated some morphologic parameters, such as microglia cell body area, microglia filaments area, microglia filaments length, number of branching points and number of segments (Supplementary Figs. S3–S4). Notably, the RC NIR group showed an increasing trend for most of the parameters measured here. Also, we observed no significant changes for HFD SHAM compared to RC SHAM, albeit there were significant differences with respect to RC NIR, thus suggesting that the molecular changes due to HFD that lead microglia activation may precede morphological modification.

### Effect of NIR on inflammatory cytokine levels in different brain areas of DIO mice

Higher pro-inflammatory cytokine expression is another important feature of neuroinflammation that occurs along with glia activation^84,85^. This characteristic is also present in both the hippocampus and cortex in animal models of obesity^86,87^, so we used q-RT-PCR to investigate whether NIR had an effect on cytokine expression levels. We analyzed mRNA levels in the hippocampus and FCTX because these two brain regions showed opposite responses to both HFD and NIR treatment in the immunohistochemistry results. Specifically, we investigated the expression levels of IL-1β and TNF-α as examples of typical pro-inflammatory cytokines and IL-10 as a typical anti-inflammatory cytokine^88^.

#### Hippocampus

**Figure 7:**
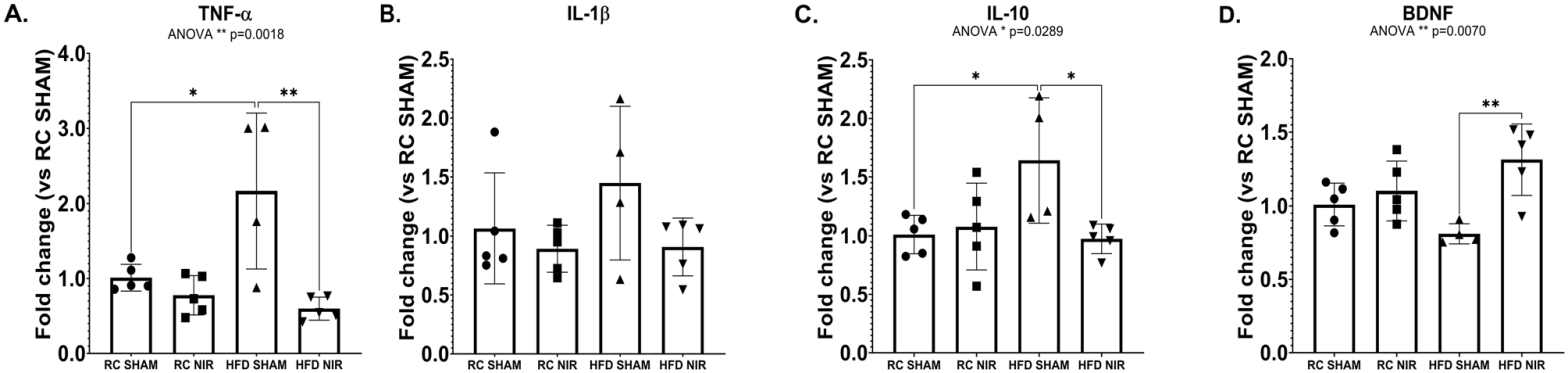
q-RT-PCR analyses of hippocampal cytokine and BDNF levels in RC- and HFD-fed mice with or without NIR. The two pro-inflammatory cytokines, TNF-α (**A**) and IL-1β (**B**), were upregulated after HFD, compared to control mice (the increase in IL-1β was not significant). NIR reduced TNF-α and IL-1β levels. The anti-inflammatory cytokine IL-10 (**C)** showed the same increasing trend as TNF-α and IL-1β. n = 4–5 animals/group; 2 replicates/animal; 2 technical replicates. Data are presented as mean ± SD. Statistical analyses: one-way ANOVA with Tukey’s post-hoc test. *p < 0.05, **p < 0.01. (**D**) Hippocampal BDNF was downregulated in HFD SHAM versus RC mice, but the decrease was not significant. NIR induced BDNF significant upregulation in HFD-fed mice, compared to untreated animals. Graphs were created with GraphPad Prism 8.4.3 and 9.3.1 software (https://www.graphpad.com/). Images and graphs were composed in Adobe Photoshop 22.2.0 (https://www.adobe.com/products/photoshopfamily.html).

HFD induced a significant increase of TNF-α (Fig. 7A) and increasing trends of IL-1β (Fig. 7B) mRNA levels compared to the RC SHAM group. NIR reduced expression levels of both cytokines to levels close to controls. Notably, the anti-inflammatory cytokine IL-10 (Fig. 7C), increased in HFD SHAM animals but returned to normal levels following NIR treatments, in line with the TNF-α data. This evidence shows that NIR can reduce pro-inflammatory cytokine expression.

#### Frontal cortex

**Figure 8:**
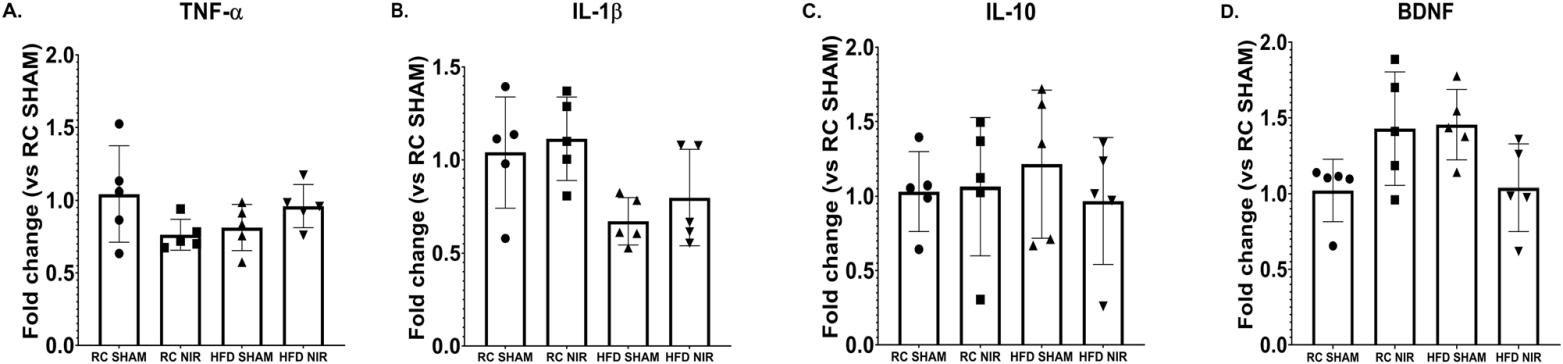
q-RT-PCR analyses of FCTX cytokine and BDNF levels in RC- and HFD-fed mice with or without NIR. There were no significant differences for TNF-α (**A**), IL-1β (**B**), IL-10 (**C**). or BDNF (**D**). n = 5 animals/group; 2 replicates/animal; 2 technical replicates. Data are presented as mean ± SD. Statistical analyses: one-way ANOVA with Tukey’s post-hoc test. Graphs were created with GraphPad Prism 8.4.3 and 9.3.1 software (https://www.graphpad.com/). Images and graphs were composed in Adobe Photoshop 22.2.0 (https://www.adobe.com/products/photoshopfamily.html).

In the FCTX, we did not observe changes in any cytokines (Fig. 8), which is in agreement with our immunofluorescence studies showing no change in glia activation with either HFD or NIR.

### Effect of NIR on NLRP3 levels in different brain areas of DIO mice

In order to determine whether the effect of NIR on reducing glia activation is accompanied by changes in other components of inflammatory machinery, we focused our attention on NLRP3, whose activation is involved in neurodegeneration and associated with increased release of IL-1β^89^. In all of the hippocampal and cortical areas that we analyzed, we did not find any significant changes among the groups for NLRP3 (Supplementary Figs. S5–6), in agreement with PCR data that did not show significant changes of IL-1β expression.

### Effect of NIR on BDNF levels in different brain areas of DIO mice

In view of the well-described downregulation of BDNF during neuroinflammation^87,90,91^, its documented role as regulator of energy metabolism and food intake^26,55,56^, and increased levels of this important neurotrophic factor after NIR treatment^65,92,93^, we measured BDNF expression levels with q-RT-PCR in our DIO mouse model. We separately analyzed BDNF mRNA levels in the hippocampus and FCTX.

#### Hippocampus

In the hippocampus (Fig. 7D) we observed a decreasing trend of BDNF after HFD. Interestingly, NIR treatment enhanced expression of this neurotrophic factor compared to HFD SHAM. This suggests that the anti-inflammatory effect of NIR might involve BDNF activation as a mechanism influencing neuroinflammation, which will be explored in future studies.

#### Frontal cortex

Unsurprisingly, BDNF did not show significant changes in the FCTX (Fig. 8D); although the statistical analysis revealed an overall significant difference (*p = 0.0308), Tukey’s multiple comparison did not show any difference among the groups, confirming that the FCTX reacts differently to the HFD challenge as observed in our immunofluorescence studies.

## Discussion

In this study, we propose targeting obesity-induced neuroinflammation as a possible strategy to prevent later cognitive decline and ultimately dementia. We focused on established features of neuroinflammation: activation of microglia and astrocytes, morphologic changes of glial cells, increased production of inflammatory cytokines^94^. Moreover, we determine the impact of NIR light on neuroinflammation in specific brain areas such as the hippocampus and cortex, that are known to be most vulnerable in neurodegenerative diseases, including AD. We hypothesized that a transcranial delivery of NIR could alleviate obesity-induced neuroinflammation by reducing glia activation and inflammatory cytokine levels in the hippocampus and cortex of DIO mice.

Obesity is a prominent risk factor for neurodegenerative diseases^95^ including AD^4^. Obesity may exacerbate AD onset and progression through several pathological mechanisms that are common to both pathologies^31,32,96^. This suggests that curbing obesity-induced CNS deficits as a possible strategy to prevent neurodegeneration and ultimately dementia. Neuroinflammation is one of the most studied mechanisms that characterizes both obesity and neurodegenerative diseases^26,46,97^. Rodent models of obesity have shown that early hypothalamic neuroinflammation develops just 2–3 days after HFD induction^98,99^, and this spreads to other brain areas such as the hippocampus^35,87,100^ and cortex^51,101,102^ when the animals are kept on HFD feeding regimens for longer times (up to 6 months). Interestingly, mixed animal models of obesity and AD show that HFD consumption and obesity worsen behavioral and cognitive deficits, synapse dysfunction, and neurodegeneration, in the setting of neuroinflammation^103–105^.

To study HFD/obesity-induced neuroinflammation we employed the DIO mouse model, which is a wild-type mouse chronically fed an HFD^106,107^. Notably, the DIO mouse model develops obesity, hyperglycemia, insulin resistance, decreased neurogenesis, impaired synaptic transmission^108,109^, and neuroinflammation. All of these events are also present in dementia, so this model affords the unique opportunity to study obesity-induced CNS deficits^110^.

### NIR alleviates HFD-induced neuroinflammation in hippocampus and POCTX, but not in FCTX

After confirming that the HFD regimen caused weight gain and hyperglycemia^106,107^, we tested whether NIR could mitigate neuroinflammation. Glia activation was investigated through FrFl-IF, which showed that HFD consumption induced microglia activation in the hippocampus and cortex, as demonstrated by elevated levels of CD68, increased astrocyte activation, and higher GFAP levels compared to RC-fed mice. We observed regional differences within these brain regions; the hippocampal CA3 and FCTX were not impacted by the HFD.

This study explored the possibility that NIR may reduce obesity-induced neuroinflammation and reduce the deleterious effects of HFD on the brain. We previously demonstrated that transcranial delivery of NIR for 4 weeks decreases synaptic vulnerability to Aβ and Tau toxic oligomers in the hippocampus and cortex of different AD-like mouse models^69,70^, while other groups demonstrated its effectiveness in reducing neuroinflammation in animal models of TBI or genetic models of Aβ-pathology^111–113^. The present findings suggest that NIR treatment could also be an effective preventative strategy for cognitive decline and neurodegeneration reducing the impact of obesity on the CNS.

Our results are consistent with the literature reporting that an HFD causes differential glial activation within different areas of the hypothalamus^114^ and hippocampus^100^, as well as from one brain area to another (including areas not included in our analyses) there are marked differences in glial activation^34,115–117^. More specifically, our results showing an increase in the astrocyte marker GFAP in the CA1, DG, and POCTX following HFD are consistent with data from the literature^33,34,82,100,115–120^, whereas studies for microglia have yielded contradictory findings. While some groups reported higher hippocampal levels of the microglial marker Iba-1 upon HFD^34,119,120^, we and others found no change in Iba-1^36,115,117^. Several studies assessing Iba-1 in the cortex showed similarities^34,114,116,117,121^ with our observation of no change following HFD, but some found increased cortical expression of Iba-1^119,120,122^.

Since we did not find significant differences in Iba-1 in the hippocampus or cortex, we investigated levels of CD68, a well-recognized marker of activated microglia^123–125^. Our results show that the HFD induced higher microglia activation in the CA1, DG, and POCTX but not in the CA3 or FCTX, which is consistent with a previous study^118^.

The differential responses to the HFD in various brain regions could be due to several reasons. It is conceivable that the different outcomes of microglia investigations depend on the specific diet regimens (in terms of type and time), as suggested by Baufeld et al.^116^. Guillemot-Legris et al. suggested that the differential CNS neuroinflammation development can be attributed to both time-dependent impairments and BBB permeability^29,31,115^, although these alterations mainly seem to involve the hypothalamus (for anatomic reasons)^46^, cerebellum^25,46,115,126^, and hippocampus^126^, but not the cortex^115^.

We found that NIR alleviates HFD-induced glia activation in the CA1, DG, and POCTX by reducing levels of the microglial marker CD68 and astrocytic marker GFAP, but there was no significant effect in the CA3 or FCTX. Still, these results suggest that this strategy may be developed to target obesity-induced neuroinflammation and possibly prevent neurodegeneration that ultimately leads to dementia. This mitigating effect on glia activation is consistent with previous studies where NIR treatment was administered a few hours or days following injury^62,66,71,111,127^, supporting the effectiveness of NIR for acute neuroinflammation. It is possible that 4 weeks of NIR treatment is insufficient to completely address the neuroinflammatory effects of 13 weeks of HFD consumption; however, the results are encouraging and warrant further investigation. It is important to emphasize that NIR was initiated following 13 weeks of HFD, when the neuroinflammation has likely become chronic. Moreover, the mice were maintained on an HFD during NIR, indicating that the treatment effectively reduces activation despite the continuous presence of stressors. This is consistent with previous investigations performed in aging mice and AD-like rodent models where NIR proved effective in alleviating neuropathology^71,72,113^.

Notably, we did not find any significant change in microglia morphology for the HFD-fed groups, even if in DG, RC NIR and HFD SHAM show significant differences in filament area, filament length, number of branching points and number of segments. We hypothesize that in our experimental conditions the HFD induces the molecular changes that drive neuroinflammation, although these are not yet accompanied by the typical morphologic changes, that characterize neuroinflammation. It is conceivable that longer diet regimens may lead to changes in microglia morphology. Likewise, it is plausible that the molecular changes due to HFD that lead microglia activation may precede morphological modification; this hypothesis seems to be supported by the observation that also HFD NIR does not show any significant morphologic change of microglia compared to the other groups, although the molecular analyses that we performed show reduction of microglia activation.

### NIR modulates inflammatory cytokine expression in DIO-mice

Neuroinflammation is characterized by increased inflammatory cytokine expression. These molecules (chemokines, ILs, TNF-α, and many others) are produced by microglia and astrocytes in the presence of pathogens or following injuries that threaten CNS homeostasis^47,83,128^. While these molecules have positive effects over short periods and facilitate repair or immune surveillance, excessive inflammatory cytokine production causes persistent inflammation and may lead to neurodegeneration^83^. HFD-induced obesity is associated with increased CNS expression of inflammatory cytokines that keeps fueling neuroinflammation, initially in the hypothalamus and later in the hippocampus and other areas^25,31,129^, exacerbating neurodegeneration^35,36^. We report that NIR can attenuate inflammatory cytokine expression, thereby alleviating a major feature of neuroinflammation.

While some studies showed increased inflammatory cytokines in the cortex of HFD-fed mice^51,101,102^, we and others did not^34,115,121,130^. This suggests that the FCTX responds differently to an HFD than the hippocampus, at least in our experimental conditions. It is also possible that longer HFD regimens would induce neuroinflammation in the FCTX. Another explanation is that structural differences between the FCTX and hippocampus may underlie differential responses to a HFD challenge, as suggested by our immunohistological data.

We observed increased hippocampal anti-inflammatory IL-10 levels with HFD, which was lowered after NIR treatment. We hypothesize that IL-10 levels may have increased as the hippocampal glia attempted to counteract the stress due to an overload of fats. A similar mechanism was proposed to explain increased IL-10 in the hypothalamus of DIO mice after 8 weeks of HFD^116^. Mice fed an HFD show an initial increase of pro-inflammatory molecules, followed by a switch to an anti-inflammatory profile during a prolonged HFD regimen, most likely in an attempt to minimize maladaptive microglia responses^116^. We observed increased hippocampal IL-10 with an HFD along with the expected increase of the pro-inflammatory cytokines (especially TNF-α), which is similar to what Han and colleagues demonstrated^131^. NIR lowers both pro-inflammatory cytokines and IL-10 levels, suggesting that hippocampal upregulation of IL-10 to cope with the chronic stimuli coming from HFD consumption is an excessive response by the activated, and likely altered, glia that NIR is able to counteract.

To further investigate the role of proinflammatory cytokines, we studied the NLRP3 inflammasome expression in brain slices; NLRP3 is involved in neuroinflammation that accompanies neurodegeneration and its activation is linked to increased IL-1β release^39,89^: our immunostaining did not show any significant difference among the various exèerimental groups, in agreement with q-RT-PCR data regarding IL-1β that did not show significant changes in its expression, thus suggesting that HFD induced-neuroinflammation is primarily driven by TNF-α.

The ability of NIR to normalize microglia activity was previously shown with in vitro and in vivo models^127,132^. Microglia cell cultures treated with NIR show higher phagocytic activity and efficiency, especially cells previously exposed to lipopolysaccharide^132^. This suggests that NIR improves the efficiency of microglia in the chronic presence of toxic molecules, and stressful conditions likely induce exaggerated and inefficient microglial responses. The NIR-induced decrease of inflammatory cytokines could be the result of greater microglia efficiency that in turn reduces neuroinflammation. In a rat model of SCI, NIR induced microglia to acquire an anti-inflammatory phenotype and reduce TNF-α secretion at 7 days post-injury^127^. This suggests that NIR triggers protective mechanisms that prevent glia activation and inflammatory cytokines release to neutralize threatening conditions that may alter homeostasis.

### NIR upregulates BDNF in DIO-mice

HFD and obesity also induce downregulation of BDNF in the hippocampus and cortex of rodents^51,53–55,57^. In addition to its known roles in synaptic plasticity and maintaining synaptic transmission and structure, BDNF is an important regulator of energy metabolism because it modulates food intake and weight gain and increases locomotor activity^26,55,56^. BDNF levels are decreased in animal models known to develop neuroinflammation^90,91,133,134^, indicating that neuroinflammatory conditions themselves may reduce levels of this neurotrophic factor. Interestingly, NIR can restore BDNF levels in in vitro^93,135^, ex vivo^92^, and in vivo^65,136^ models of neuroinflammation. Following HFD, we observed a decreasing trend for hippocampal BDNF with immunostaining analyses and cytokine evaluations. NIR dramatically increased BDNF levels in this brain region, but we did not observe significant group differences in the FCTX. Once again, we observed different outcomes in the hippocampus and FCTX, further suggesting that these two areas respond differently to HFD consumption. However, it is important to note that we cannot exclude the possibility that various HFD protocols might yield different outcomes. Whether NIR-induced upregulation of BDNF directly decreases neuroinflammation or is an effect of the diminished neuroinflammation remains to be established. It is also possible that NIR activates protective mechanisms somehow involving BDNF, which should be examined in future studies.

### Final considerations

This study did not explore mechanistic pathways to ascertain the anti-inflammatory effect of NIR; rather we sought to establish whether NIR can be a preventative treatment for conditions known to pose serious threats to CNS integrity^59,60,137^.

^50,60,111,138,139^ Previous studies have shown that NIR decreases brain levels of the main anti-inflammatory cytokines (IL-1β and TNF-α) and reduces glia activation in different models of TBI and AD^59,61,92,93,111–113,127,132,135,136,140–142^. Likewise, NIR upregulates BDNF in rodent injury models and in animal models that develop neuroinflammation, suggesting that it may provide neuronal and synaptic protection^92,93,135,136,142^. Given the paramount importance of BDNF in maintaining synapse function and integrity, we hypothesize that NIR-induced upregulation in the hippocampus may restore or improve synaptic transmission and neuronal plasticity, with beneficial effects on memory and learning processes. Indeed, our previous work showed that NIR ameliorates cognitive impairment in hTau mice models^69^ while decreasing toxic oligomer concentrations in hippocampal and cortical synaptosomes^69,70^.

In light of our results, we speculate that NIR may be further developed as a protective treatment for subjects with obesity, diabetes, or metabolic syndrome who are at high risk of developing dementia. Several recent studies and clinical trials have successfully used transcranial NIR delivery in patients with AD and other neurologic and neurodegenerative disorders such as depression, PD and TBI^58,61,142,143^, and it has been shown to improve cognitive performance in normal subjects^144–146^. We consider this approach a valid strategy as a preventative treatment for dementia and AD, especially for individuals who are obese and/or insulin-resistant/diabetic and therefore at high risk of developing cognitive dysfunction later in life.

## Conclusion

Our study demonstrates that NIR can reverse neuroinflammation associated with obesity, one of the key events that risk of AD. NIR also enhances hippocampal BDNF expression that could induce neuroprotective mechanisms to counteract the metabolic challenge. We also show that HFD/obesity-induced neuroinflammation differentially affects distinct brain areas, suggesting that some regions are more vulnerable to the challenge of an HFD. Moreover, we propose a novel approach aimed at reducing obesity-induced neuroinflammation and related effects on brain function. Overall, the present results indicate that transcranial NIR delivery has therapeutic potential as a non-invasive approach to protect against obesity-induced CNS deficits that are known to occur during the AD neuropathological cascade. These findings underscore the need for clinical testing of NIR as a preventative treatment in people with obesity-related risk of AD.

## Supplementary Information

Supplementary Information.

## Data Availability

The datasets used and/or analyzed during the current study are available from the corresponding author on reasonable request.

## Abbreviations

AD: Alzheimer’s disease
ANOVA: Analysis of variance
AUC: Area under the curve
Aβ: Amyloid β
BBB: Blood–brain barrier
BDNF: Brain derived neurotrophic factor
BSA: Bovine serum albumin
CA1: Cornu ammonis 1
CA3: Cornu ammonis 3
CD68: Cluster of differentiation-68
cm^2^: Square centimeters
CNS: Central nervous system
DAPI: 4′,6-diamidino-2-phenylindole
DG: Dentate gyrus
DIO: Diet-induced obese
FCTX: Frontal cortex
FrFl: Free floating
GFAP: Glial fibrillar acidic protein
HFD: High-fat diet
Iba-1: Ionized calcium-binding adapter-1
IF: Immunofluorescence
IL-10: Interleukin-10
IL-1β: Interleukin-1beta
IPGTT: Intraperitoneal glucose tolerance test
J: Joule
mRNA: Messenger ribonucleic acid
NGS: normal goat serum
NIR (light): Near infra-red (light)
nm: Nanometers
PBM: Photobiomodulation
PBS: Phosphate buffer solution
PD: Parkinson’s disease
POCTX: Parietal/occipital cortex
q-RT-PCR: Quantitative real time polymerase chain reaction
RC: Regular chow
ROS: Reactive oxygen species
SCI: Spinal cord injury
SD: Standard deviation
T2DM: Type 2 diabetes mellitus
TBI: Traumatic brain injury
TBS: Tris buffer solution
TLR: Toll-like receptor
TNF-α: Tumor necrosis factor-alpha
USDA: United States Department of Agriculture
UTMB: University of Texas Medical Branch
W: Watts
