# Supplementary material for: Near-infrared light reduces glia activation and modulates neuroinflammation in the brains of diet-induced obese mice
Source: Sci Rep. 2022 Jun 27;12:10848. doi: 10.1038/s41598-022-14812-8 (PMC9237037; doi:10.1038/s41598-022-14812-8)
Supplement: Supplementary file 1 — Supplementary Information. [file 41598_2022_14812_MOESM1_ESM.pdf]

Near-Infrared Light Reduces Glia Activation and Modulates Neuroinflammation in the Brains of Diet-Induced Obese Mice

Salvatore Saieva[1,2] and Giulio Taglialatela[1, *]

[1]Mitchell Center for Neurodegenerative Diseases, Department of Neurology, University of Texas Medical Branch, Galveston, TX, USA

[2]Department of Neuroscience, Cell Biology and Anatomy, University of Texas Medical Branch, Galveston, TX, USA

[*] Corresponding author. E-mail address: gtaglial@utmb.edu

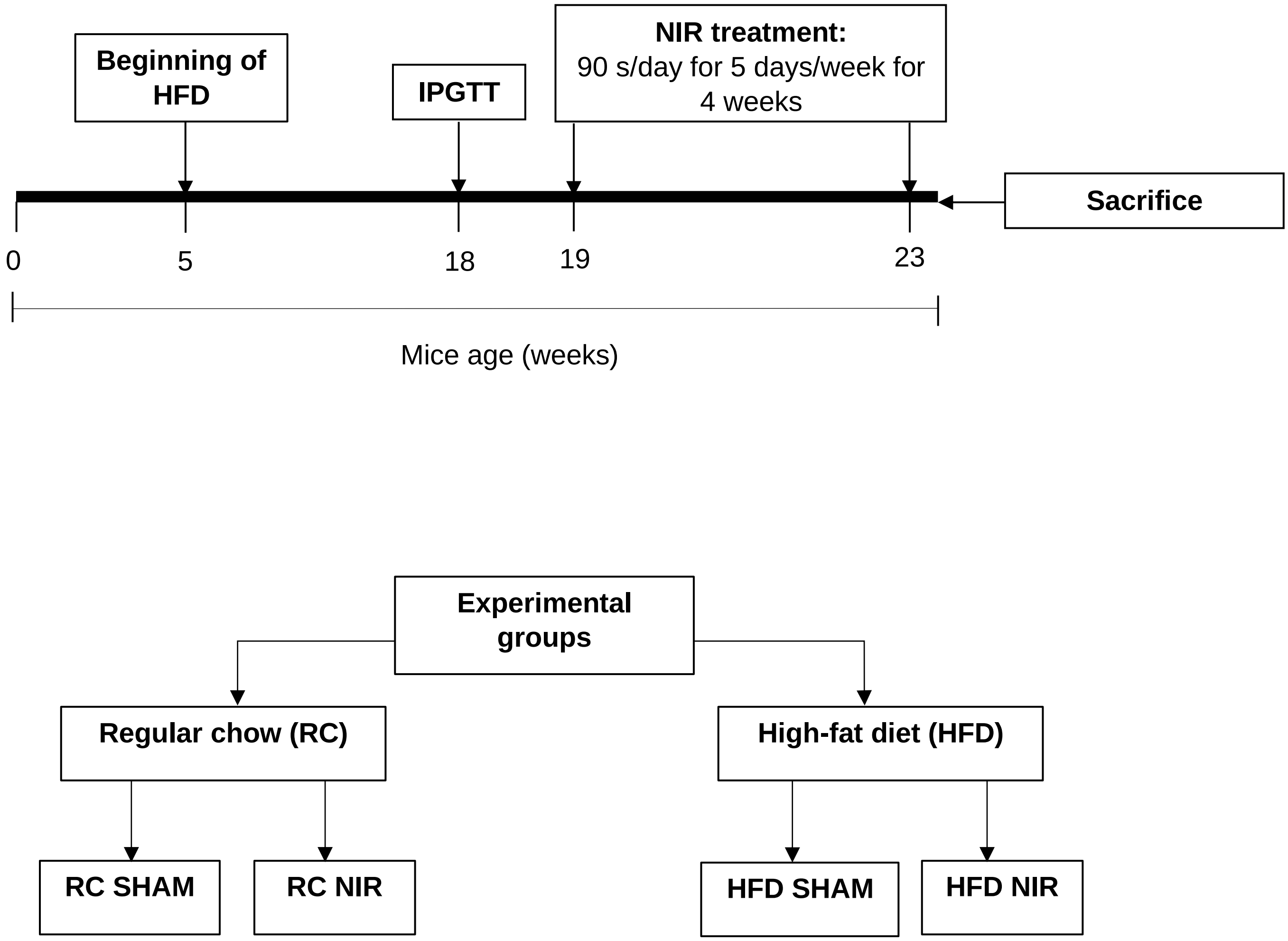

Supplementary Figure S1. Experimental design. Timeline with experimental procedures (*upper panel*) and description of experimental groups (*lower panel*). Panels created with Word-Microsoft 365 (https://www.microsoft.com/en-us/microsoft-365/word).

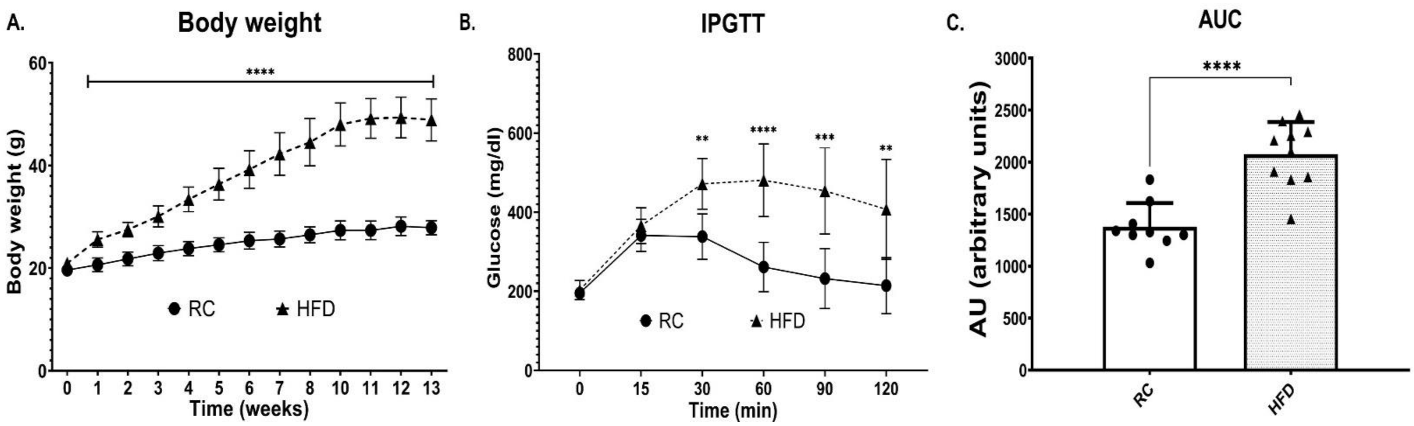

Supplementary Figure S2. Assessment of body weight and blood glucose levels after 13 weeks of high-fat diet (HFD). (A) Weekly evaluations of body weight show weight gain for mice on an HFD compared to RC-fed mice. Data are presented as mean±SD. Each dot represents the average of 10 animals/group. Statistical analyses: two-way ANOVA with Sidak's post-hoc test. ****p<0.0001. **(B)** Following 12 weeks of HFD, mouse blood glucose levels were evaluated through intraperitoneal glucose tolerance test (IPGTT). After 5 h of fasting and an intraperitoneal glucose injection, HFD induced higher blood glucose levels during the 2 h after glucose administration, compared to controls. Graph presented as mean±SD. Each dot represents the mean of 9-10 animals/group per time point. Statistical analyses: two-way ANOVA with Sidak's post-hoc test. **p<0.01, ***p<0.001, ****p<0.0001. **(C)** Area under the curve (AUC) of glucose vs. time. Graph presented as mean±SD. Statistical analyses: one-tailed t-test with Mann-Whitney post-hoc test. Each dot represents a single animal. Graphs were created with GraphPad Prism 8.4.3 and 9.3.1 software (https://www.graphpad.com/). Images and graphs were composed in Adobe Photoshop 22.2.0 (https://www.adobe.com/products/photoshopfamily.html).

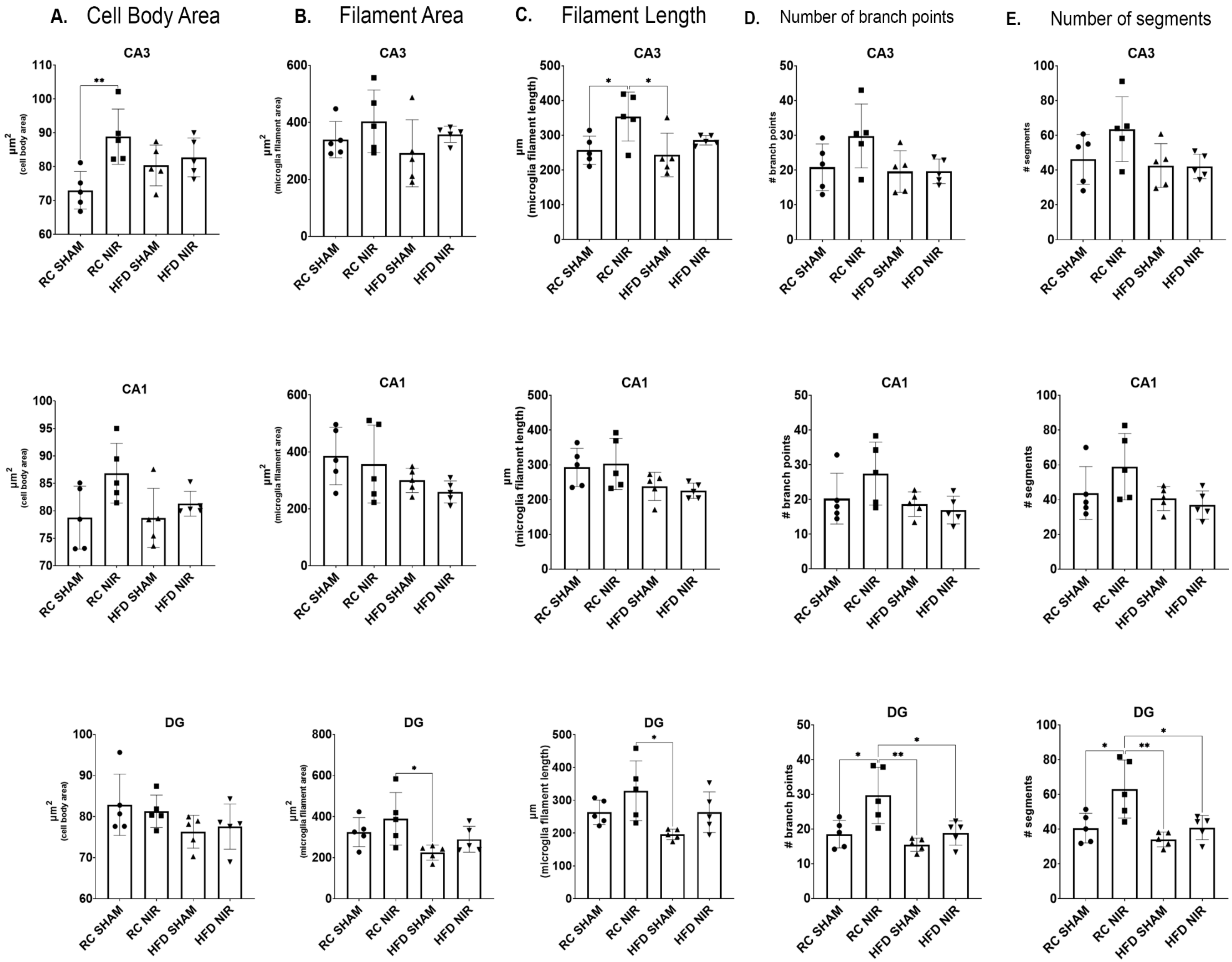

A. Cell Body Area
B. Filament Area
C. Filament Length
D. Number of branch points
E. Number of segments
CA3
CA1
DG
μm2 (cell body area)
μm2 (microglia filament area)
μm (microglia filament length)
branch points
segments
RC SHAM
RC NIR
HFD SHAM
HFD NIR

Supplementary Figure S3. Morphologic analyses of microglia in hippocampus. In column **A.** the graphs for cell body area are reported, in column **B.** the filament area, in column **C.** the filament length, in column **D.** the number of branch points, and in column **E.** the number of segments. In the *upper row* the data from CA3 are represented, in the *middle row* the data from CA1, in the *lower row* the data from DG. There are no significant changes between HFD-groups and RC-groups, although in DG HFD SHAM vs. RC NIR groups show significant differences in filament area, filament length, number of branch points and number of segments. We hypothesize that HFD, in our conditions, does not induce morphologic alterations but, rather, molecular changes that lead to microglia activation, as well as NIR acts on these molecular players in HFD animals. Data are presented as mean±SD. Statistical analyses: one-way ANOVA with Tukey's post-hoc test. *$p<0.05$, **$p<0.01$. Graphs were created with GraphPad Prism 8.4.3 and 9.3.1 software (https://www.graphpad.com/). Images and graphs were composed in Adobe Photoshop 22.2.0 (https://www.adobe.com/products/photoshopfamily.html).

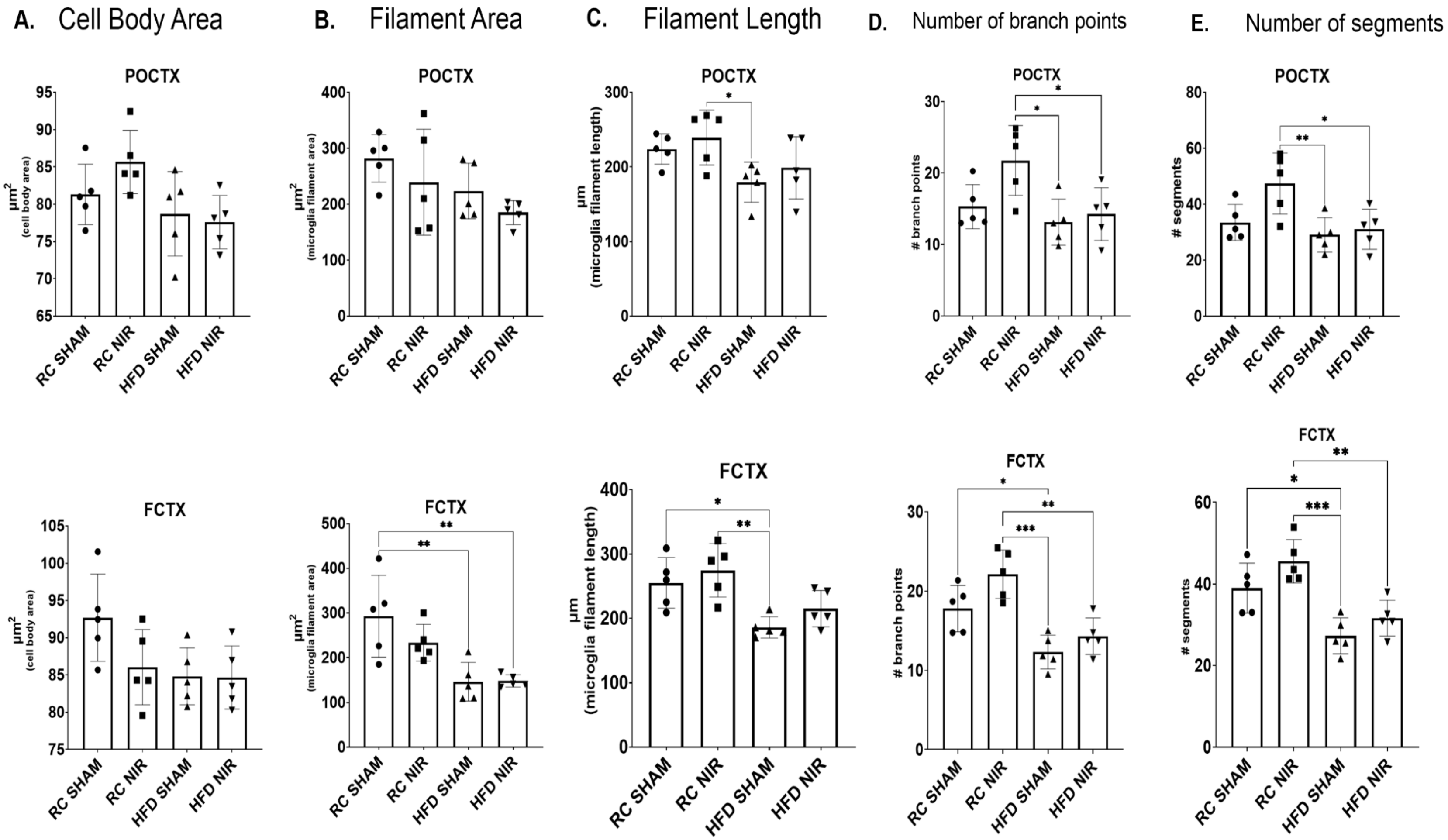
A. Cell Body Area
B. Filament Area
C. Filament Length
D. Number of branch points
E. Number of segments
POCTX
FCTX
µm2 (cell body area)
µm2 (microglia filament area)
µm (microglia filament length)
branch points
segments
RC SHAM
RC NIR
HFD SHAM
HFD NIR

Supplementary Figure S4. Morphologic analyses of microglia in cortical areas. In column **A.** the graphs for cell body area are reported, in column **B.** the filament area, in column **C.** the filament length, in column **D.** the number of branch points, and in column **E.** the number of segments. In the *upper row* the data from POCTX are represented, in the *lower row* the data from FCTX. Also in the cortical areas, we observe siginifcant changes between RC NIR and HFD SHAM for some parameters, although this data seems to support our hypothesis that the molecular changes may precede the morphological ones. Data are presented as mean±SD. Statistical analyses: one-way ANOVA with Tukey's post-hoc test. *p<0.05, **p<0.01. Graphs were created with GraphPad Prism 8.4.3 and 9.3.1 software (https://www.graphpad.com/). Images and graphs were composed in Adobe Photoshop 22.2.0 (https://www.adobe.com/products/photoshopfamily.html).

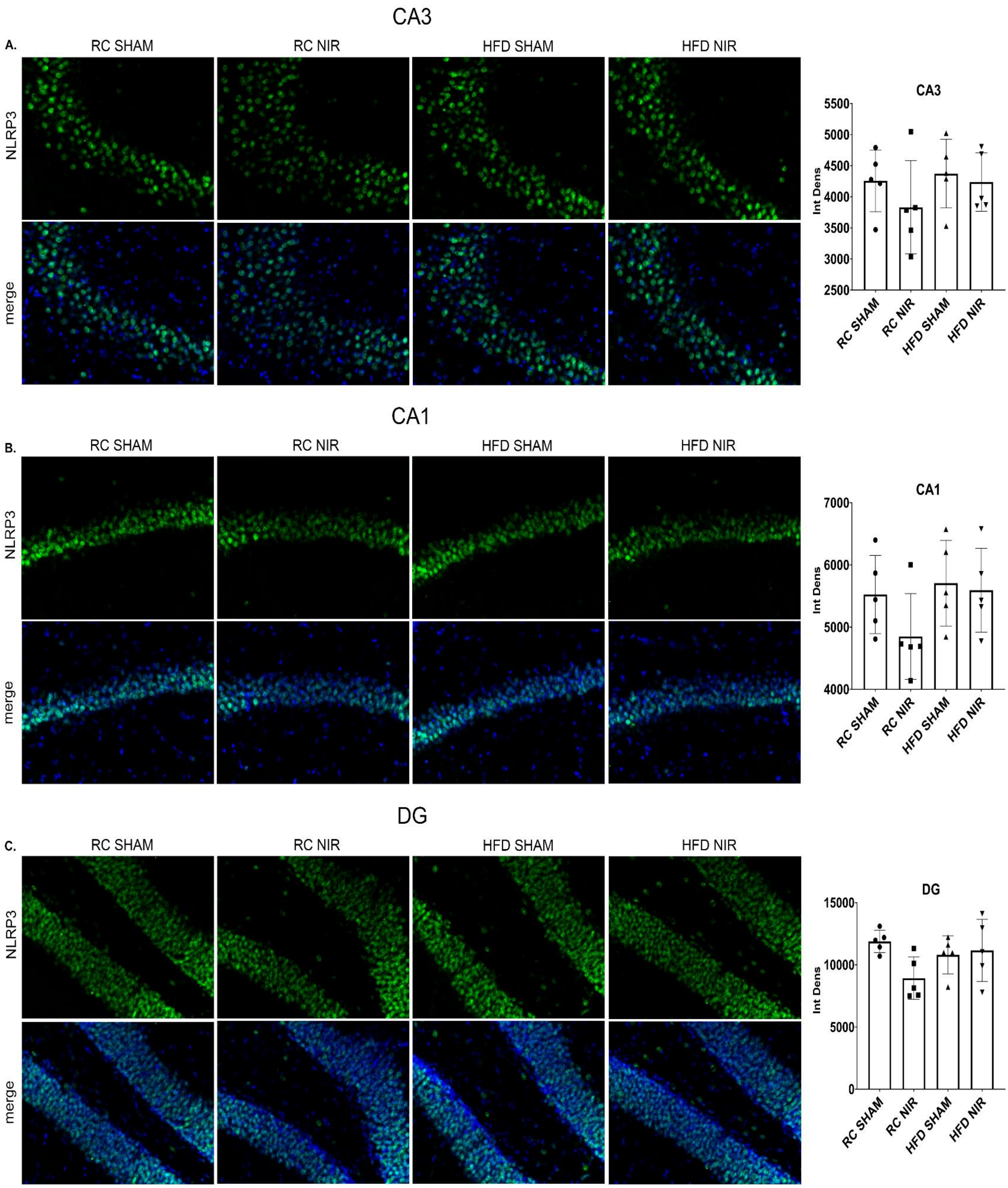
CA3
A.
RC SHAM
RC NIR
HFD SHAM
HFD NIR
NLRP3
merge
CA3
Int Dens
5500
5000
4500
4000
3500
3000
2500
RC SHAM
RC NIR
HFD SHAM
HFD NIR
CA1
B.
RC SHAM
RC NIR
HFD SHAM
HFD NIR
NLRP3
merge
CA1
Int Dens
7000
6000
5000
4000
RC SHAM
RC NIR
HFD SHAM
HFD NIR
DG
C.
RC SHAM
RC NIR
HFD SHAM
HFD NIR
NLRP3
merge
DG
Int Dens
15000
10000
5000
0
RC SHAM
RC NIR
HFD SHAM
HFD NIR

Supplementary figure S5. NLRP3 expression in the hippocampus of RC- and HFD-fed mice with or without NIR. We do not observe any significant changes among the groups in CA3 (**A.**), CA1 (**B.**) and DG (**C.**). Magnification: 40X. Image results of Z-stacks with pitch of 2.0 of µm. n= 5 animals/group. Data are presented as mean±SD. Each point is the average of 6 images/animal from 2 technical replicates. Statistical analyses: one-way ANOVA with Tukey's post-hoc test. Single images were generated with Keyence BZ-X800 Analyzer (www.keyence.com). Graphs were created with GraphPad Prism 8.4.3 and 9.3.1 software (https://www.graphpad.com/). Images and graphs were composed in Adobe Photoshop 22.2.0 (https://www.adobe.com/products/photoshopfamily.html).

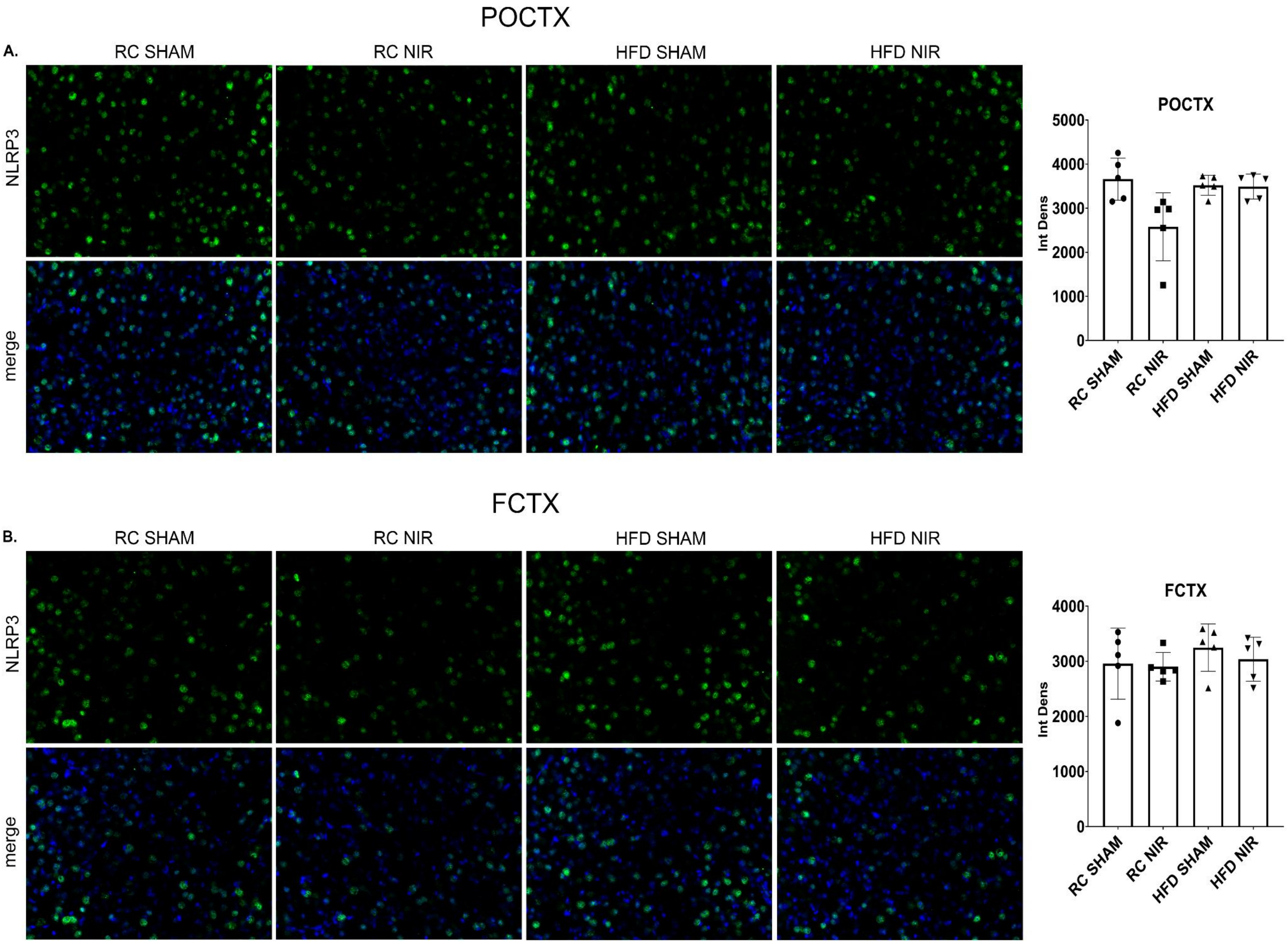
POCTX
A.
RC SHAM
RC NIR
HFD SHAM
HFD NIR
NLRP3
merge
POCTX
5000
4000
3000
2000
1000
0
Int Dens
RC SHAM
RC NIR
HFD SHAM
HFD NIR
FCTX
B.
RC SHAM
RC NIR
HFD SHAM
HFD NIR
NLRP3
merge
FCTX
4000
3000
2000
1000
0
Int Dens
RC SHAM
RC NIR
HFD SHAM
HFD NIR

Supplementary figure S6. NLRP3 expression in the cortical areas of RC- and HFD-fed mice with or without NIR. Also, in the cortical areas, no change is reported between the analyzed groups, in both POCTX (**A.**) and FCTX (**B.**). Magnification: 40X. Image results of Z-stacks with pitch of 2.0 of μm. n= 5 animals/group. Data are presented as mean±SD. Each point is the average of 6 images/animal from 2 technical replicates. Statistical analyses: one-way ANOVA with Tukey's post-hoc test. Single images were generated with Keyence BZ-X800 Analyzer (www.keyence.com). Graphs were created with GraphPad Prism 8.4.3 and 9.3.1 software (https://www.graphpad.com/). Images and graphs were composed in Adobe Photoshop 22.2.0 (https://www.adobe.com/products/photoshopfamily.html).

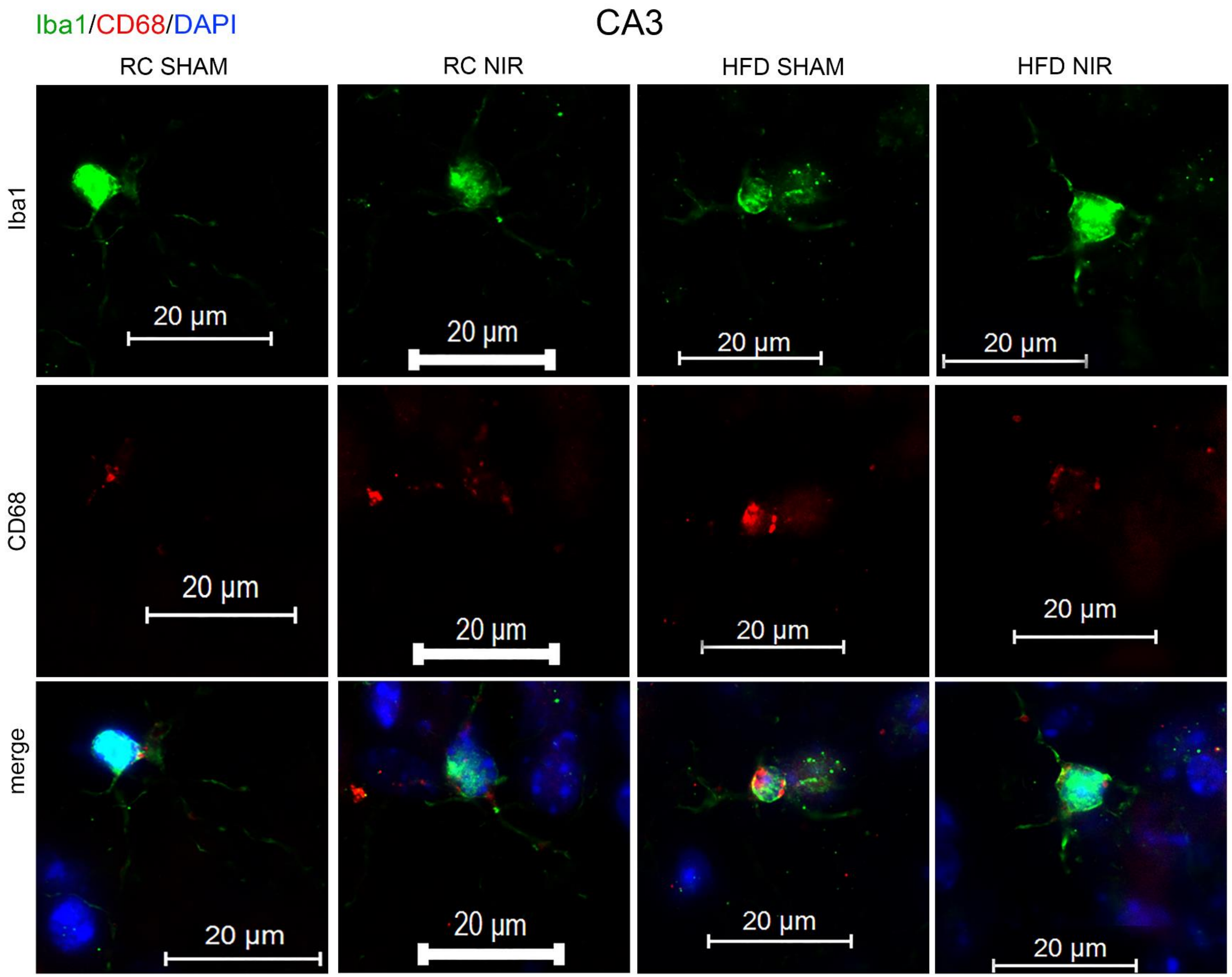

Iba1/CD68/DAPI
CA3
RC SHAM
RC NIR
HFD SHAM
HFD NIR
Iba1
CD68
merge
20 µm

Supplementary Figure S7: Enlargements of microglia cells in CA3 at 100X magnification. Original images were cropped, and a single microglia cell was highlighted. Single images were generated with Keyence BZ-X800 Analyzer (www.keyence.com). Images and graphs were composed in Adobe Photoshop 22.2.0 (https://www.adobe.com/products/photoshopfamily.html).

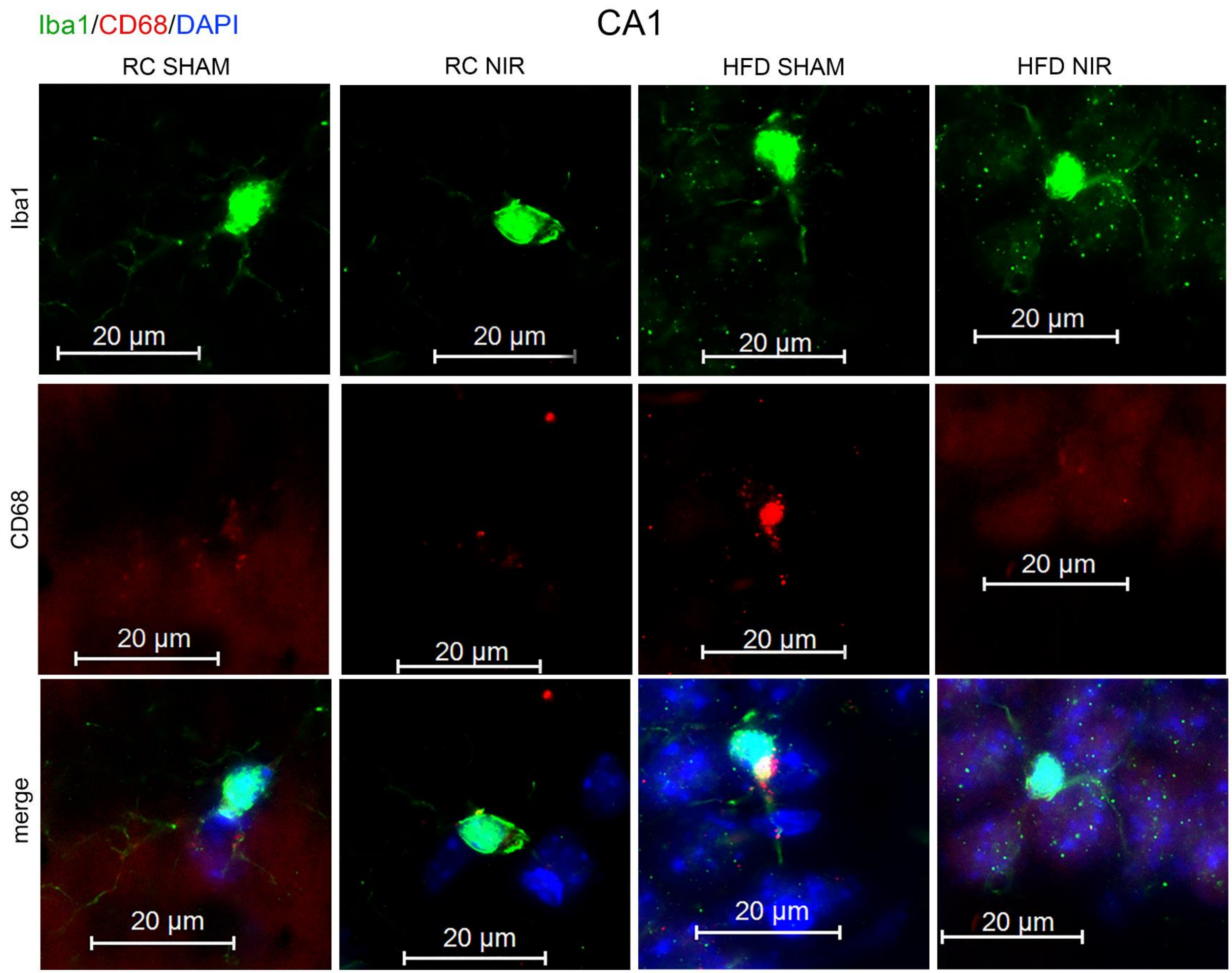
Iba1/CD68/DAPI
CA1
RC SHAM
RC NIR
HFD SHAM
HFD NIR
Iba1
CD68
merge
20 µm

Supplementary Figure S8: Enlargements of microglia cells in CA1 at 100X magnification. Original images were cropped, and a single microglia cell was highlighted. Single images were generated with Keyence BZ-X800 Analyzer (www.keyence.com). Images and graphs were composed in Adobe Photoshop 22.2.0 (https://www.adobe.com/products/photoshopfamily.html)

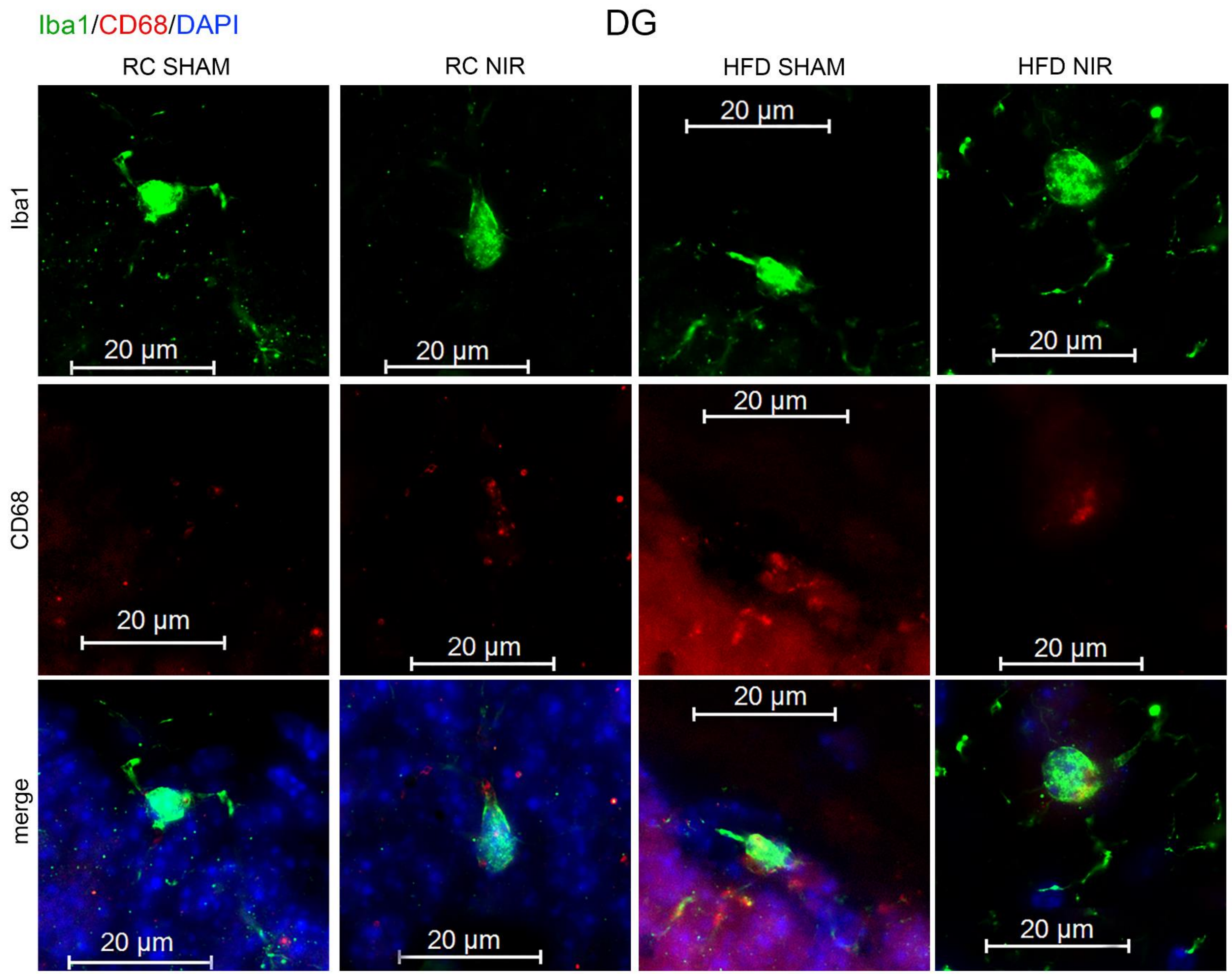
Iba1/CD68/DAPI
DG
RC SHAM
RC NIR
HFD SHAM
HFD NIR
Iba1
CD68
merge
20 μm

Supplementary Figure S9: Enlargements of microglia cells in DG at 100X magnification. Original images were cropped, and a single microglia cell was highlighted. Single images were generated with Keyence BZ-X800 Analyzer (www.keyence.com). Images and graphs were composed in Adobe Photoshop 22.2.0 (https://www.adobe.com/products/photoshopfamily.html).
